# Niclosamide, but not ivermectin, inhibits anoctamin 1 and 6 and attenuates inflammation of the respiratory tract

**DOI:** 10.1007/s00424-023-02878-w

**Published:** 2023-11-18

**Authors:** Jiraporn Ousingsawat, Raquel Centeio, Rainer Schreiber, Karl Kunzelmann

**Affiliations:** Physiological Institute, University of Regensburg, Germany University Street 31, 93053 Regensburg, Germany

**Keywords:** Anoctamin 1, Anoctamin 6, Inflammation, Asthma, Cystic fibrosis, COVID-19

## Abstract

Inflammatory airway diseases like cystic fibrosis, asthma and COVID-19 are characterized by high levels of pulmonary cytokines. Two well-established antiparasitic drugs, niclosamide and ivermectin, are intensively discussed for the treatment of viral inflammatory airway infections. Here, we examined these repurposed drugs with respect to their anti-inflammatory effects in airways *in vivo* and *in vitro*. Niclosamide reduced mucus content, eosinophilic infiltration and cell death in asthmatic mouse lungs *in vivo* and inhibited release of interleukins in the two differentiated airway epithelial cell lines CFBE and BCi-NS1.1 *in vitro*. Cytokine release was also inhibited by the knockdown of the Ca^2+^-activated Cl^−^ channel anoctamin 1 (ANO1, TMEM16A) and the phospholipid scramblase anoctamin 6 (ANO6, TMEM16F), which have previously been shown to affect intracellular Ca^2+^ levels near the plasma membrane and to facilitate exocytosis. At concentrations around 200 nM, niclosamide inhibited inflammation, lowered intracellular Ca^2+^, acidified cytosolic pH and blocked activation of ANO1 and ANO6. It is suggested that niclosamide brings about its anti-inflammatory effects at least in part by inhibiting ANO1 and ANO6, and by lowering intracellular Ca^2+^ levels. In contrast to niclosamide, 1 µM ivermectin did not exert any of the effects described for niclosamide. The present data suggest niclosamide as an effective anti-inflammatory treatment in CF, asthma, and COVID-19, in addition to its previously reported antiviral effects. It has an advantageous concentration–response relationship and is known to be well tolerated.

**Supplementary Information:**

The online version contains supplementary material available at 10.1007/s00424-023-02878-w.

## Introduction

Inflammatory airway diseases are characterized by high levels of pulmonary cytokines. Two antiparasitic compounds, niclosamide and ivermectin, are discussed as repurposed drugs for the treatment of inflammatory airway disease in asthma, cystic fibrosis and COVID-19 [12, 21, 55, 61, 83]. Niclosamide is a common anthelminthic effective against tapeworm. It is also used as a molluscicide and for the treatment of schistosomiasis (bilharziasis) [3]. Uncoupling of oxidative phosphorylation in worms and snails is discussed as its main antiparasitic mechanism [27, 80]. We reported pronounced suppression of intracellular Ca^2+^ signalling by niclosamide and inhibition of the Ca^2+^ activated Cl^−^ channel anoctamin 1 (ANO1, TMEM16A) as well as the phospholipid scramblase anoctamin 6 (ANO6, TMEM16F) [7, 12, 15, 19, 44]. Both may contribute to its anthelminthic effect. The drug ivermectin is also well established as an antiparasitic compound to treat infestations by scabies infection and many other parasites [46]. Its antiparasitic effect is due to the activation of glutamate-activated Cl^−^ channels specific to nonvertebrates, although it also finds a few additional targets in vertebrate cells [46].

Both niclosamide and ivermectin have antiviral properties and are currently evaluated in clinical trials for the use in coronavirus disease 2019 (COVID-19) (clinicalTrials.gov) [1, 4, 61, 72]. Niclosamide compromises acidic pH in endolysosomes and lowers cytosolic Ca^2+^ concentrations, which is thought to contribute to these properties [25, 34, 41, 62, 72, 82]. In contrast, ivermectin reduces viral infection by inhibiting importins, soluble transport receptors that are essential for the nucleo-cytoplasmic transit of substrates [17, 51, 59]. Niclosamide and ivermectin are suggested to inhibit ANO1 currents and ANO6-mediated phospholipid scrambling that is crucial for formation of multinucleated airway epithelial cells (syncytia) and blood clotting observed during SARS-CoV-2 infection and after vaccination against COVID-19 [9, 18, 71].

ANO1 and ANO6 are inhibited by niclosamide, which also inhibits mucus secretion and airway constriction [12, 20, 21, 55, 57]. We therefore asked in the present study whether ivermectin may equally inhibit anoctamins, affect lung functions and exert anti-inflammatory effects. Here, we report anti-inflammatory effects of niclosamide at low concentrations comparable to plasma concentrations detected during oral medication. In contrast, none of these effects, including the lack of inhibition of anoctamins, was observed with ivermectin, suggesting a different molecular mechanism of action for ivermectin.

## Materials and methods

### Animals and treatments

All animal experiments complied with the general guidelines for animal research, in accordance with the UK Animals Act 1986, and associated guidelines, and EU Directive 2010/63/EU for animal experiments. The experiments were approved by the local Ethics Committee of the Government of Unterfranken/Wurzburg/Germany (AZ: 55.2–2532-2–677) and adhered to the ARRIVE guidelines. Allergen challenge of mice has been described in Schreiber et al. [68]. In brief, C57BL/6 J mice (*n* = 12) were sensitized to ovalbumin (OVA; Sigma-Aldrich, St. Louis, MO, USA) by intraperitoneal (I.P.) injection of 100 µg OVA in 100 µl aluminium hydroxide gel adjuvant (InvivoGen, San Diego, CA, USA) on days 0 and 14. From days 21 to 23, mice were anesthetized and challenged to OVA by intratracheal (I.T.) instillation of 50 µg OVA in 100 µl saline. Control mice (*n* = 4) were sham sensitized with the adjuvant aluminium hydroxide gel and challenged to saline by I.T. instillation. Thirty micromolar niclosamide or 30 µM ivermectin, both dissolved in 100 µl saline, or 100 µl saline only (control) was applied by I.T. instillation for consecutive 5 days. The mice were first challenged, and after completing the challenge, the mice received niclosamide (*n* = 4) or ivermectin (*n* = 4). After the completed 5th day of receiving the drugs (or control saline), the animals were humanly killed.

### Immunocytochemistry TUNEL-assay, secreted hCLCA1

Mouse lung tissues were collected for semi-quantitative RT-PCR analysis of anoctamin expression. Mouse airways were fixed by transcardial perfusion and lung perfusion by tracheal instillation via tracheostomy of fixative solution containing 4% paraformaldehyde (PFA) in PBS. Tissues were left in fixative solution overnight and embedded in paraffin the next day. Five-micrometer cuts were deparaffinized, stained with standard Alcian blue solution, and counterstained with Nuclear Fast Red solution (Sigma-Aldrich, St. Louis, MO, USA). After the dehydration and clearing steps, whole mouse lungs or sections were mounted in DePeX mounting medium (SERVA Electrophoresis, Heidelberg, Germany). Stainings were assessed by light microscopy. Stitching microscopy was used to analyze whole mouse lungs. For analysis of eosinophils, sections were dewaxed, rehydrated, and stained according to Pappenheim [64].

Terminal deoxynucleotidyl transferase dUTP nick end labelling (TUNEL) was performed in a PFA fixed tissue, embedded in paraffin. The DeadEnd Fluorometrie TUNEL system (Promega, Mannheim, Germany) was used according to the manufacturer’s instructions. To obtain hCLCA1‐conditioned media, HEK293 cells were seeded at 500,000 cells per 6 well and transfected the next day with empty pcDNA3.1 (mock) or hCLCA1 plasmids, using standard protocols for Lipofectamine 3000 (Invitrogen, Carlsbad, California, USA). After 6 h, the medium was changed to serum‐free media (Gibco/Thermo Fisher Scientific, Waltham, Massachusetts USA), and 24 h later the media, control or enriched in secreted hCLCA1 protein, were collected. hCLCA1-conditioned media were administered to OVA‐challenged animals by I.T. instillation in the absence or presence of niclosamide or ivermectin. CLCA1 was detected using rabbit anti-mouse Clca3 antibody (ab46512, Abcam, Berlin, Germany); Nuclei were counterstained with 5 µM Hoe33342 (Thermo Fisher Scientific, Darmstadt, Germany). Immunofluorescence was detected with an Axio Observer microscope equipped with Axiocams 503 mono, ApoTome.2 and ZEN 3.0 (blue edition) software (Zeiss, Oberkochen, Germany). Stitching microscopy (image stitching) was used to combine multiple photographic images with overlapping fields of view to produce high-resolution images of whole mouse lungs and airways, using a motorized Axio Observer and Zen software [63].

### Cell lines

All cells were grown at 37 °C in a humidified atmosphere with 5% (v/v) CO2. Culture conditions for CFBE cells have been described earlier [12]. In brief, CFBE/CFTR and CFBE/parental cells were grown in MEM with Earle’s Salts with L-glutamine medium (Capricorn Scientific, Ebsdorfergrund, Germany) supplemented with 10% FBS. BCi-NS1.1 cells (kindly provided by Prof. R. Crystal, Weill Cornell Medical College, New York, USA) were cultured in supplemented bronchial epithelial cell growth medium (BEGM; Lonza, Basel, Switzerland) [78]. Cells were either grown on plastic or were grown on permeable supports for up to 30 days (Snapwell #3801, Corning, New York, USA) in an air/liquid interface (ALI). THP-1 macrophages were grown in RPMI1640 medium, supplemented with 10% FBS, 1% penicillin/streptomycin. Jurkat T-cells were cultured in RPMI1640 supplemented with 10% FBS, 2 mM L-glutamine, and 1% penicillin/streptomycin. U-DCS dendritic cells were kindly provided by Prof. Dr. Kevin Mellert and Prof. Dr. Peter Möller (lnstitute of Pathology, Albert-Einstein-AIlee 23, D-89070 Ulm, Germany) [54]. Cells were grown in Iscove/RPMI medium supplemented with 10% fetal calf serum, 2 mM L-glutamine, 100 U/ml penicillin, 100 µg/ml streptomycin, and insulin–transferrin–sodium selenite supplement (ITS, Roche, Mannheim, Germany). In order to study the release of IL-8, U-DCS cells were stimulated for 24 h with 0.5 ml maturation medium (10 ng/ml TNF-α, 10 ng/ml IL-1β, 15 ng/ml IL-6 and 1 µg/ml PGE2). Human embryonic kidney 293 (HEK293) cells were grown in DMEM media supplemented with 2 mM L-glutamine. HEK293 cells were transfected with cDNA encoding human anoctamin 1 or anoctamin 6 [70].

### RT-PCR, siRNA, western blot

For semi-quantitative RT-PCR analysis of anoctamin expression, total RNA was isolated using NucleoSpin RNA II columns (Macherey–Nagel, Düren, Germany). Total RNA (1 µg/50 µl reaction) was reverse-transcribed using a random primer (Promega, Mannheim, Germany) and M-MLV reverse transcriptase RNase H Minus (Promega, Mannheim, Germany). Each RT-PCR reaction contained sense and antisense primers for mouse anoctamins as described earlier [70], 0.5 µl cDNA and GoTaq Polymerase (Promega, Mannheim, Germany). After 2 min at 95 °C, cDNA was amplified 25 cycles for 30 s at 95 °C, 30 s at 56 °C and 1 min at 72 °C. PCR products were visualized by loading on Midori Green Xtra (NIPPON Genetics, Dueren, Germany) containing agarose gels and analyzed using ImageJ (NIH, Bethesda, MA, USA). For the knockdown of ANO1 or ANO6, cells were transfected with siANO1 (5-CCUGUACGAAGAGUGGGCACGCUAU-3) or siANO6 (5-CCUCCAUCAUCAGCUUUAUAAUUAU-3; both from Invitrogen, Carlsbad, CA^2+^, USA) using standard protocols for Lipofectamine 3000 (Invitrogen, Carlsbad, CA, USA), scrambled siRNA (Silencer® Select Negative Control siRNA #1, Ambion, Austin, TX, USA). Western blots were performed as described in [20].

### Cytokine analysis by ELISA

Cytokine release was assessed in CFBE airway epithelial cells. Release of IL-2, IL-6, IL-8, and CLCA1 was detected using quantikine colorimetric sandwich ELISA kits (R&D systems, Wiesbaden-Nordenstadt, Germany) before and after incubation with LPS or IL-13 (both 24 h) and in the absence or presence of various concentrations of niclosamide or ivermectin. To that end, supernatants were collected, particulates were removed by centrifugation and assays were performed immediately according to the protocol of the manufacturer. Signals were detected using a microplate reader (NOVOstar; BMG Labtech, Offenburg, Germany).

### LDH-release, propidium iodide and FACS

Supernatants were collected from cells and were assessed using the CytoTox96® non-radioactive cytotoxicity assay (Promega) at a wavelength of 490 nm. Percentage of LDH release was calculated as 100 × (experimental LDH-spontaneous LDH)/(maximum LDH release-spontaneous LDH). Cell death was also detected by propidium iodide permeabilization. For flow cytometry, cells were collected using accutase (Capricorn Scientific, Ebsdorfergrund, Germany), washed with cold Dulbecco’s PBS (DPBS) and centrifuged at 500 g and 4 °C for 10 min. Subsequently, cells were resuspended in a 100-µl annexin binding buffer containing 5 µl annexin V-FITC and 2.5 µl 7-aminoactinomycin D (7-AAD; BioLegend, Koblenz, Germany). Fluorescence-activated cell sorting (FACS) analyses were performed in the Annexin V standard binding buffer (BioLegend, San Diego, CA, USA) containing 10 mM Hepes, 140 mM NaCl and 2.5 mM CaCl. For each measurement, at least 10,000 cells were analyzed by flow cytometry at 37 °C (BD AccuriTM C6, St. Ives, UK). 7-AAD, a non-permeant dye, was used to identify cells with plasma membrane leakage. Freshly isolated macrophages were stained with propidium iodide staining to detect RSL3/erastin-induced cell death.

### Measurements of intracellular pH and intracellular Ca^2+^

For intracellular pH measurements, cells were mounted under the microscope and perfused with HCO_3_^−^/CO2 solution (mmol/l, NaCl 118.75; KH2PO4 0.4; K2HPO4 1.6; glucose 5; MgSO4 1; Ca-gluconate 1.3, NaHCO3 25; bubbled with 95% O2/5% CO2). Transport was stimulated with Cl^−^ free HCO_3_^−^/CO2 solution (bubbled with 95% O2/5% CO2). ΔpH was taken as a measure of Cl^−^/HCO_3_^− ^− antiport. Experimental procedures, acquisition of images and data analysis were described recently [8]. Measurement of cytosolic Ca^2+^ changes was performed as described recently [16]. In brief, HEK293T, IHKE-1 and cortical primary cells were loaded with 2 µM Fura-2, AM (BIOZOL, Eching, Germany) in OptiMEM (Gibco, Thermo Fisher Scientific) with 0.02% Pluronic F-127 (Invitrogen, Thermo Fisher Scientific,) in ringer solution (mmol/l, NaCl 145; KH2PO4 0,4; K2HPO4 1,6; Glucose 5; MgCl2 1; Ca^2+^-gluconat 1,3) for 1 h at room temperature. Fluorescence was detected in cells perfused with Ringer’s solution at 37 °C using an inverted microscope (Axiovert S100, Zeiss, Germany) and a high-speed polychromator system (VisiChrome, Puchheim, Germany). Fura-2 was excited at 340/380 nm, and the emission was recorded between 470 and 550 nm using a CCD camera (CoolSnap HQ, Visitron Systems, Germany). [Ca2 +]_*i*_ was calculated from the 340/380 nm fluorescence ratio after background subtraction. The formula used to calculate [Ca^2+^]_*i*_ was [Ca^2+^]_*i*_ = Kd × (*R* − *R*_min_)/(*R*_max_ − *R*) × (Sf2/Sb2), where *R* is the observed fluorescence ratio. The values *R*_max_ and *R*_min_ (maximum and minimum ratios) and the constant Sf2/Sb2 (fluorescence of free and Ca^2+^-bound Fura-2 at 380 nm) were calculated using 2 µmol/l ionomycin (Biomol GmbH, Hamburg, Germany) and 5 mmol/l EGTA to equilibrate intracellular and extracellular Ca^2+^ in intact Fura-2-loaded cells. The dissociation constant for the Fura-2•Ca^2+^ complex was taken as 224 nmol/l [36]. Control of experiment, imaging acquisition, and data analysis were done with the software package Meta-Fluor (Universal imaging, USA).

### Patch clamp

Cells were patch clamped when grown on coated glass coverslips. Coverslips were mounted in a perfused bath chamber on the stage of an inverted microscope (IM35, Zeiss) and kept at 37 °C. Patch pipettes were filled with a cytosolic-like solution containing (in mM) KCl 30, K-gluconate 95, NaH2PO4 1.2, Na2HPO4 4.8, EGTA 1, Ca-gluconate 0.758, MgCl2 1.03, D-glucose 5, and ATP 3; pH 7.2. The intracellular (pipette) Ca^2+^ activity was 0.1 µM. The bath was perfused continuously with standard bicarbonate-free Ringer’s solution (in mM NaCl 145, KH2PO4 0.4, K2HPO4 1.6, glucose 5, MgCl 2 1, Ca^2+^-gluconate 1.3) at a rate of 4 ml/min. Patch pipettes had an input resistance of 3–5 MΩ, and whole-cell currents were corrected for serial resistance. Currents were recorded using a patch clamp amplifier EPC9 and PULSE software (HEKA, Lambrecht, Germany) as well as the Chart software (AD Instruments, Spechbach, Germany). Cells were stimulated with 1 µM ATP in the absence and presence of TRAM34. In regular intervals, membrane voltage (Vc) was clamped in steps of 20 mV from − 100 to + 100 mV from a holding voltage of − 100 mV. The current density was calculated by dividing whole-cell currents by cell capacitance.

### Materials and statistical analysis

Except of special compounds and kits listed above, general chemicals were from Sigma-Aldrich (St. Louis, Missouri, USA). BCi-NS1.1 cells were kindly provided by Prof. R. Crystal, Weill Cornell Medical College, New York, USA. U-DCS dendritic cells were kindly provided by Prof. Dr. Kevin Mellert and Prof. Dr. Peter Möller (lnstitute of Pathology, Albert-Einstein-AIlee 23, D-89070 Ulm, Germany). Data are shown as individual traces/representative images and/or as summaries with mean values ± SEM, with the respective number of experiments given in each figure legend. For statistical analysis, paired or unpaired Student’s *t* test or ANOVA was used where appropriate. A *p*-value of < 0.05 was accepted as a statistically significant difference.

## Results

### Niclosamide but not ivermectin reduced mucus content, eosinophilic infiltration and cell death in asthmatic mouse lungs

**Fig. 1:**
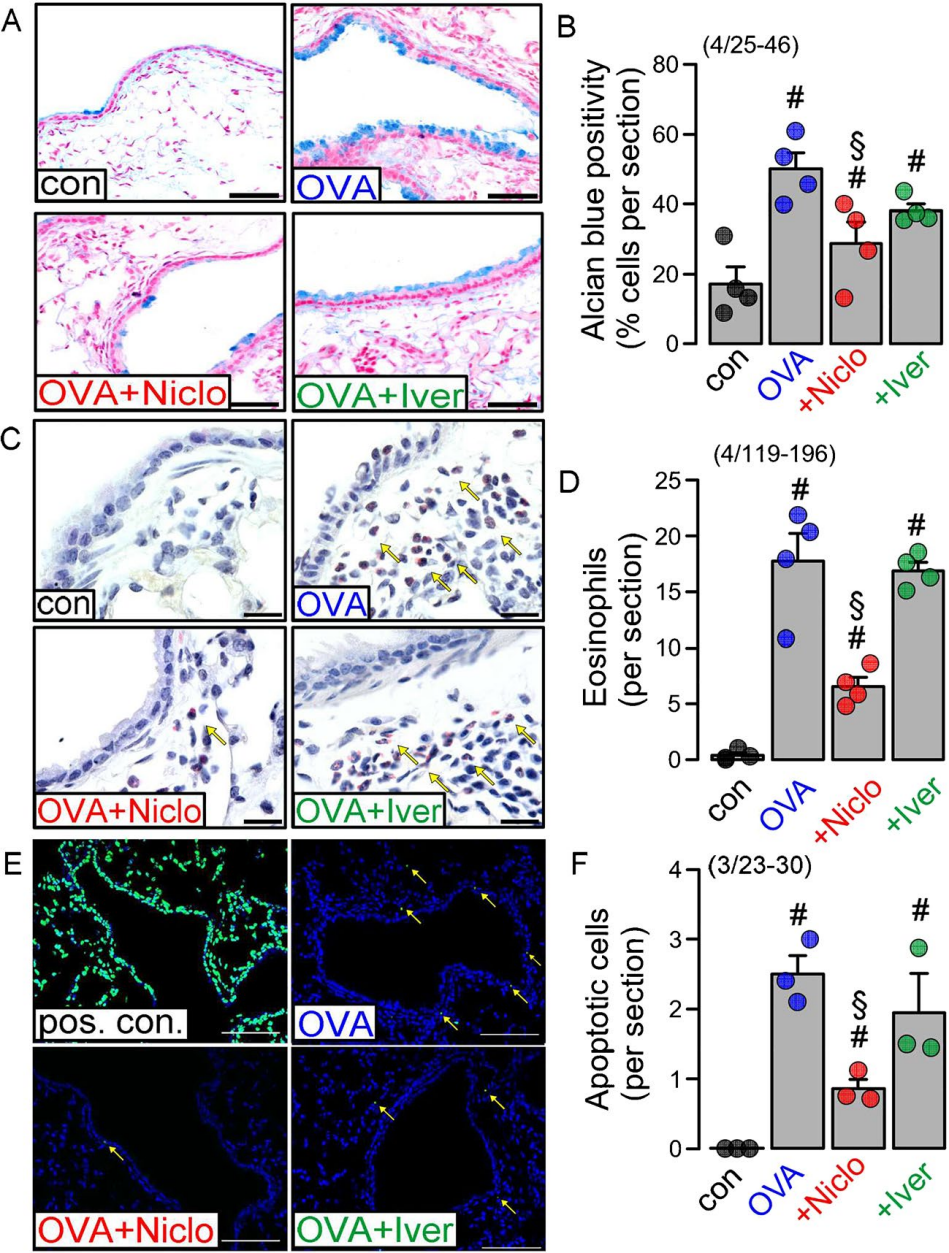
Niclosamide but not ivermectin reduces mucus content, eosinophilic infiltration and cell death in asthmatic mouse lungs. **A** Airways from a sham-treated mouse (con), an ovalbumin-treated asthmatic mouse (OVA), and asthmatic mice treated with Niclo or Iver (OVA + drug; tracheal instillation of 100 µl of 30 µM Niclo or Iver dissolved in 100 µl saline for 5 days). Alcian blue staining indicates strong reduction of mucus by treatment with Niclo but not Iver. Bars = 100 µm. **B** Summary of Alcian blue stainings. **C** Eosinophils (interstitial cells with a red Pappenheim eosin staining; yellow arrows) present in airways of the different cohorts. Bars = 100 µm. **D** Summary of eosinophil numbers. **E** TUNEL positive cells in DNase 1-treated lung sections (pos. con.), and in lungs from mice treated with OVA, OVA + Niclo and OVA + Iver, respectively (yellow arrows). Bars = 300 µm. **F** Number of apoptotic cells was significantly reduced by Niclo but not by Iver. Mean ± SEM (number of animals/number of analyzed sections). Number sign (#) indicates significant increase compared with con (*p* < 0.05; ANOVA). Section sign (§) indicates significant difference compared with OVA (*p* < 0.05; ANOVA)

Effects of niclosamide (Niclo) and ivermectin (Iver) on inflamed airways were analyzed *in vivo* by intratracheal instillation of either compound (30 µM Niclo or 30 µM Iver dissolved in 100 µl saline applied for 5 consecutive days) to asthmatic (ovalbumin sensitized; OVA) mice. Sham-treated animals received 100 µl saline without drugs. No weight loss was observed during application of the drugs. OVA-treated mice demonstrated impressive goblet cell metaplasia, which was significantly attenuated by Niclo, but not by Iver (Fig. 1A, B). Eosinophilic infiltration in lungs of asthmatic mice was significantly reduced only by Niclo (Fig. 1C, D). Cell death of airway epithelial cells was assessed in TUNEL assays. TUNEL-positive (apoptotic) cells were found in airways of asthmatic mice, but not in healthy control airways, similar to a previous report [43]. The number of apoptotic cells was reduced in Niclo-treated animals but not in Iver-treated animals (Fig. 1E, F). The data suggest mucostatic and anti-inflammatory effects by Niclo, which, however, are not induced by Iver.

### Accumulation of CLCA1 in mouse airway epithelial cells in the presence of Niclo is possibly due to inhibition of CLCA1-secretion

**Fig. 2:**
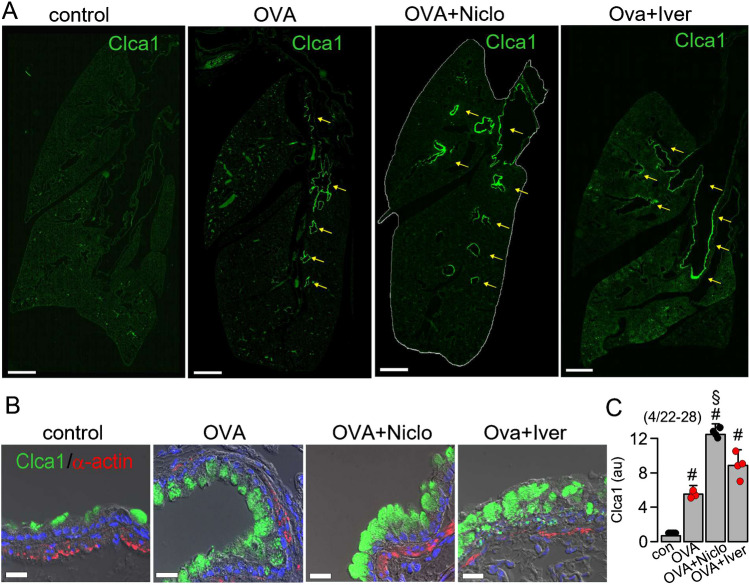
Niclosamide but not ivermectin inhibits release of CLCA1 from epithelial cells of mouse lungs. **A** Staining of CLCA1 in airways (yellow arrows) of whole lung sections of con, OVA, OVA + Niclo and OVA + Iver mice, respectively. Bar = 1 mm. **B** clca1 and smooth muscle α-actin from con, OVA, OVA + Niclo and OVA + Iver mice. Nuclei (blue) were stained by Hoe33342. Bar = 20 µm. **C** Summary of CLCA1 stainings under the different conditions. Mean ± SEM (number of animals/number of sections analyzed). Number sign (#) indicates significant increase in fluorescence intensity when compared to con (*p* < 0.05; ANOVA). Section sign (§) indicates significant difference compared with OVA (*p* < 0.05; ANOVA)

CLCA1 (chloride channel accessory 1) is an important regulator of the Ca^2+^-activated Cl^−^ channel anoctamin 1 (ANO1) [42]. An N-terminal fragment resulting from the self-proteolysis of CLCA1 is a secreted protein with protease activity, which directly binds to ANO1, thereby stabilizing ANO1 in the plasma membrane [65]. CLCA1 also drives mucus production, upregulates proinflammatory genes in Th-2 mediated airway inflammation and CLCA1 itself can act as a signalling molecule [26, 31, 42]. Previously named mouse CLCA3 is now designated CLCA1 as it is the direct ortholog to human CLCA1 [56]. Staining of CLCA1 in mouse whole lung sections showed upregulation of CLCA1 in asthmatic mice, which was not reduced by treatment with Niclo or Iver (Fig. 2A). In contrast, Niclo (but not Iver) enhanced staining of intracellular CLCA1 (Fig. 2B, C). As shown below, Niclo inhibits ANO1 and ANO6 by direct and indirect mechanisms [12, 55]. It attenuates intracellular Ca^2+^ signalling by affecting receptor-mediated release of Ca^2+^ from the endoplasmic reticulum (ER) store [19], thereby inhibiting exocytosis [7, 12, 20, 67]. Niclo may therefore also compromise cellular release of CLCA1. To that end, we examined release of CLCA1 from IL-13-exposed CFBE human airway epithelial cells and found that Niclo as well as the knockdown of ANO1 or ANO6 inhibited the release of CLCA1 (Fig. S1). These results are in line with our previous findings, showing a role of both anoctamins for exocytosis [20]. Along this line, a pronounced increase in CLCA1-staining was observed previously in airways of mice lacking expression of ANO1 [13].

### CLCA1 can operate as a cytokine-like signal molecule whose release is inhibited by niclosamide

Independent of the presence of the metalloprotease domain, CLCA1 was shown to induce the release of proinflammatory cytokines such as IL-1β, IL-6, TNF-α and IL-8 [26]. To further examine the signalling function of CLCA1, we applied secreted hCLCA1 (100 µl for 5 consecutive days) to mouse airways via intratracheal instillation. Secreted hCLCA1 was obtained from conditioned supernatant of HEK293 cells heterologously expressing hCLCA1. Supernatant from mock transfected cells served as control [22]. We showed previously that secreted hCLCA1 augments mucus production and mucus secretion and induces plasma membrane expression of Ano1 in mouse airways and ANO1 currents in airway epithelial cells [22]. These results suggest that secreted human CLCA1 is active in mouse tissues. Moreover, we found evidence that hCLCA1 activates ANO1 expressed endogenously in mouse M1 cells [14, 76]. hCLCA1 present in conditioned media from hCLCA1-expressing HEK293 cells [22] significantly increased Ca^2+^-dependent whole cell currents (activated by 1 µM ATP) from 2.04 ± 0.46 nA (mock; *n* = 4) to 3.63 ± 0.52 nA (hCLCA1; *n* = 5; *p* < 0.05, unpaired *t* test).

**Fig. 3:**
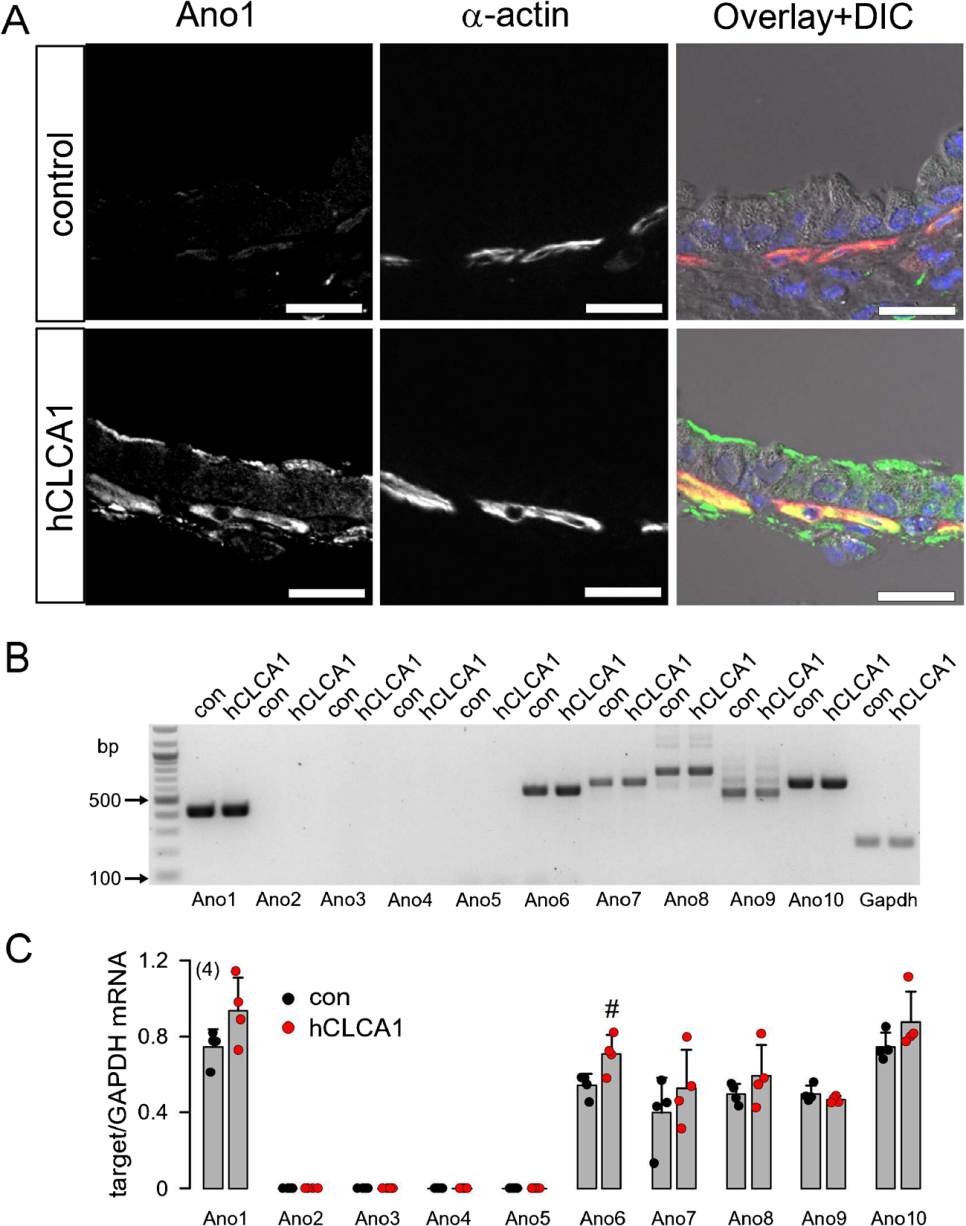
Luminal application of hCLCA1 induces membrane expression of Ano1 in airway epithelial and smooth muscle cells. **A** Ano1 and α-actin in mouse airways exposed to control media (from mock transfected HEK293 cells) or media containing secreted hCLCA1 (each 100 µl/24 h exposure). hCLCA1 induced pronounced expression of Ano1 in mouse airway epithelial cells and in the airway smooth muscle. Nuclei (blue) were stained by Hoe33342. Bars = 20 µm. **B**, **C** Gel and summary from semiquantitative RT-PCR analysis of expression of the ten different anoctamin proteins (Ano1-Ano10) in mouse airways exposed to control media (con) or hCLCA1-containing media. Mean ± SEM (number of reactions). Number sign (#) indicates significant increase in expression by hCLCA1 (*p* < 0.05; unpaired *t* test)

Here, we show that hCLCA1 induced the pronounced expression of Ano1 not only in the airway surface epithelium, but also in submucosal airway smooth muscle (ASM) cells (Fig. 3A). This finding indeed suggests a signalling function of CLCA1 in mouse airways. mRNA was isolated from mouse airways, and semiquantitative RT-PCR analysis of the expression of anoctamins was performed. No increase in Ano1-mRNA by CLCA1 could be detected, confirming the stabilization of ANO1 in the plasma membrane, rather than transcriptional upregulation, as described previously [65] (Fig. 3B, C). Interestingly, we found that hCLCA1 itself can induce CLCA1 release (Fig. S2). Thus, luminally applied CLCA1 does induce cytokine release by airway epithelial cells [26], which in turn upregulates Ano1 in submucosal ASM cells.

### Niclosamide and knockdown of ANO1, but not ivermectin, inhibit release of inflammatory cytokines in CFBE airway epithelial cells

**Fig. 4:**
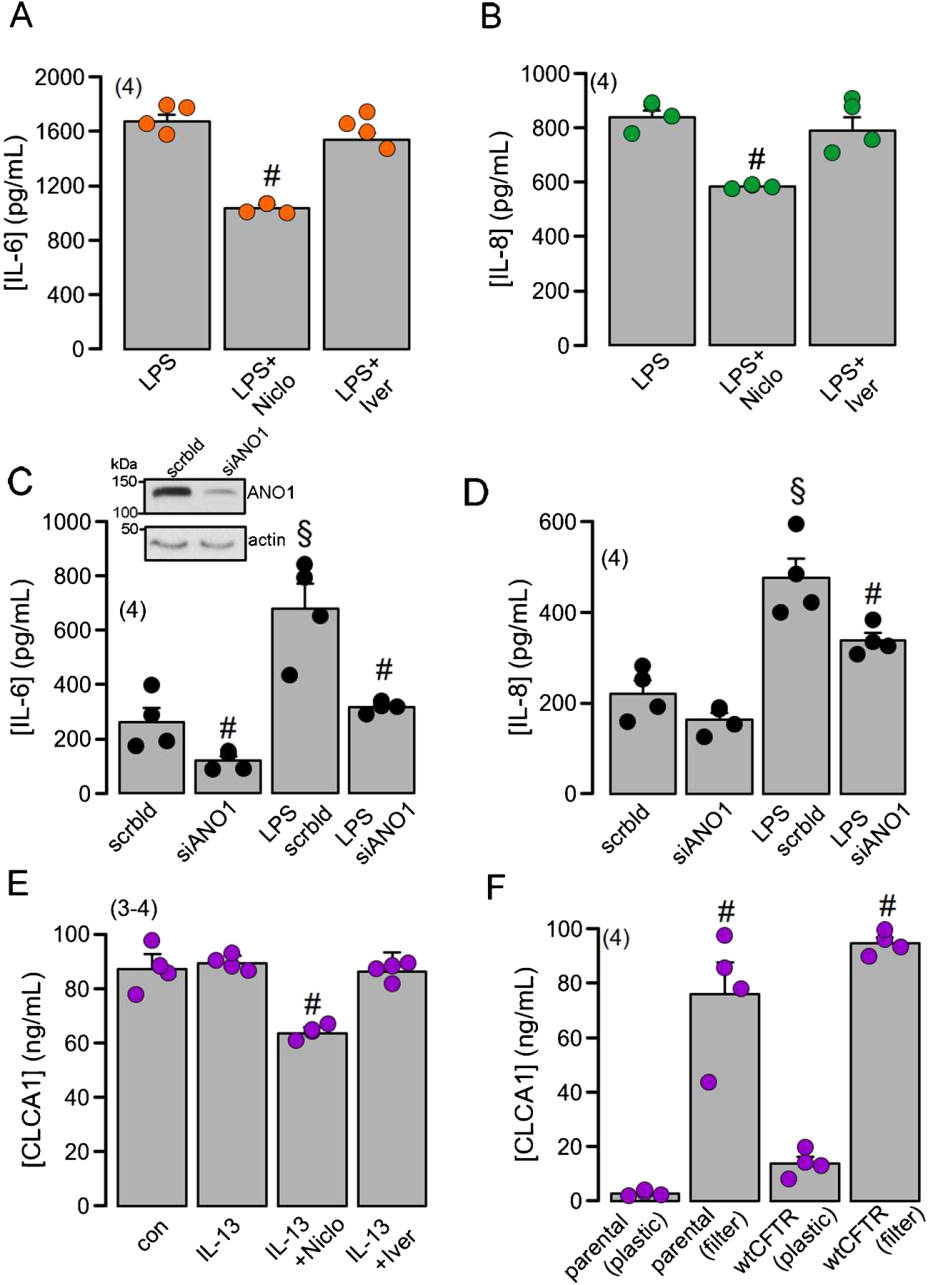
Niclosamide and ANO1-knockdown, but not ivermectin, inhibit release of the inflammatory cytokines IL-6 and IL-8 from CFBE airway epithelial cells. **A**, **B** Release of IL-6 and IL-8 from filter-grown CFBE cells in the absence or presence of Niclo or Iver (each 200 nM), respectively. **C**, **D** Knockdown of ANO1 (siANO1) inhibited release of IL-6 and IL-8 from plastic-grown CFBE cells, when compared with cells treated with scrambled RNA (scrbld). **E** High basal release of CLCA1 (not further stimulated by 20 ng/ml IL-13) was partially inhibited by Niclo but not Iver. **F** Demonstration that basal CLCA1-release from CFBE cells depends on growth conditions and independent of CFTR-expression. Mean ± SEM (number of experiments). Number sign (#) indicates significant inhibition by Niclo and knockdown of ANO1 (*p* < 0.05; unpaired *t* test). Section sign (§) indicates significant increase of cytokine release by LPS (*p* < 0.05; unpaired *t* test)

Due to animal ethics regulations, we were unable to analyze cytokines by bronchoalveolar lavages. We therefore examined in filter-grown human CFBE airway epithelial cells stably expressing wtCFTR (CFBE/CFTR cells) whether Niclo and Iver affect the release of IL-6, IL-8 and CLCA1 [47]. Basal constitutive release of both IL-6 and IL-8 was surprisingly high and was not further enhanced by LPS (100 ng/ml; 24 h) (Fig. 4A, B). Nevertheless Niclo (but not Iver) inhibited the release of both cytokines (both at 200 nM). We examined the effect of siRNA-knockdown of ANO1 on LPS-induced cytokine release. These experiments were performed in plastic-grown CFBE/CFTR cells, as they show lower basal cytokine release, and cytokine release could be further enhanced upon LPS stimulation (Fig. 4C, D). Knockdown of ANO1 significantly reduced the release of IL-6 and IL-8 [7]. As shown above for IL-6 and IL-8, filter-grown CFBE/CFTR cells also showed spontaneous release of CLCA1, which was not further enhanced by IL-13 (20 ng/ml; 24 h) but was inhibited by Niclo (Fig. 4E). A previous study reported the impact of growth conditions of CFBE cells on cytokine release recently [52]. We also found that the spontaneous release of CLCA1 entirely depends on the growth conditions of CFBE cells, but independent of the expression of CFTR (Fig. 4F). Therefore, the inhibition of cytokine release by Niclo and the role of ANO1 was re-examined in the more differentiated cell line BCi-NS1.1 (see Fig. 4).

### Niclosamide and knockdown of ANO1 and ANO6 inhibit release of inflammatory cytokines in BCi-NS1.1 airway epithelial cells

**Fig. 5:**
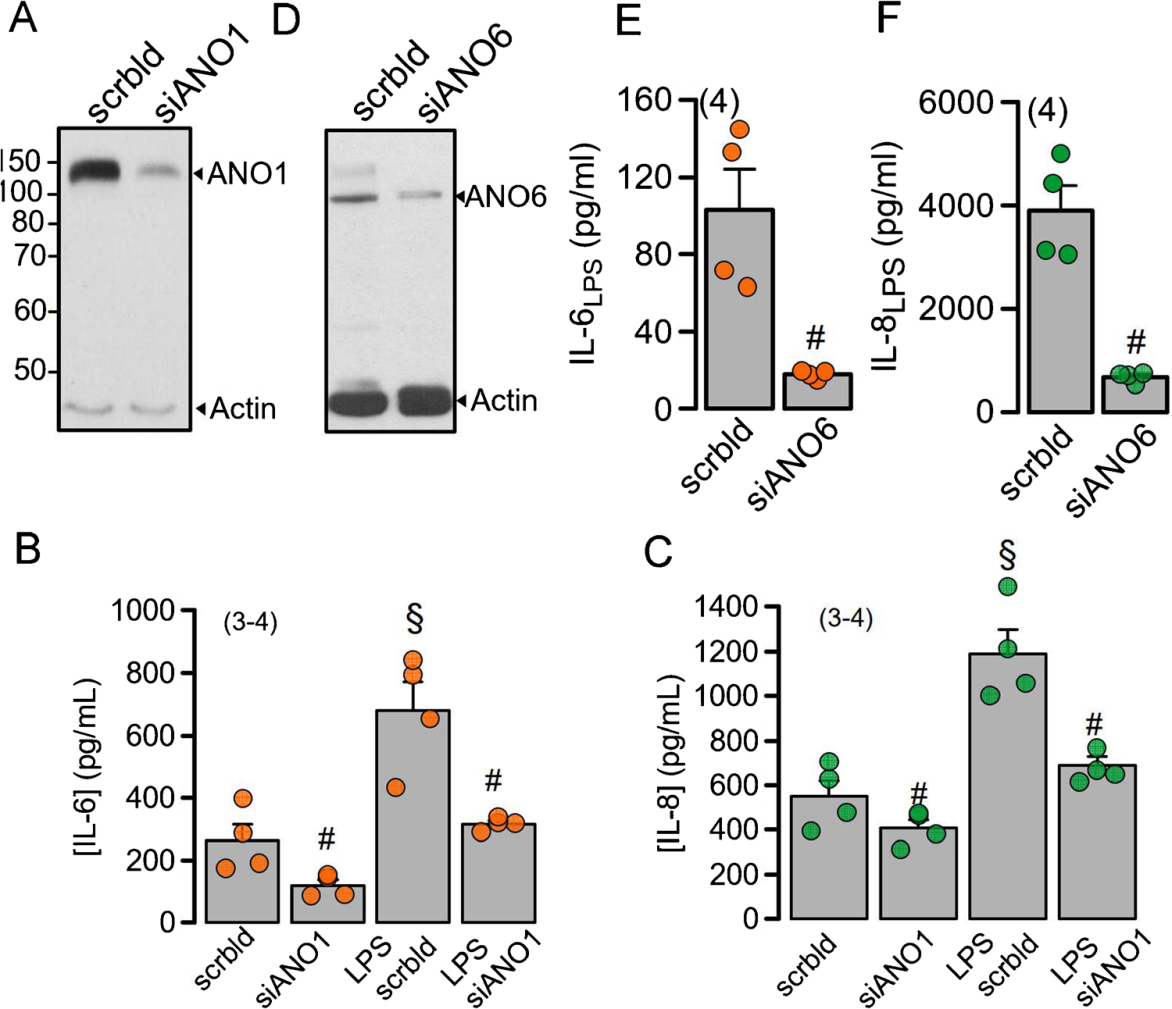
siRNA-knockdown of ANO1 or ANO6 inhibits release of IL-6 and IL-8 by human airway epithelial cells. **A**, **D** Western blots indicating knockdown of ANO1 and ANO6 by siRNA. Blots were performed in triplicates. **B**, **C** Release of IL-6 and IL-8 in the absence or presence of LPS (100 ng/ml) (24 h). Knockdown of ANO1 (siANO1) inhibits release of IL-6 and IL-8, but not scrambled RNA (scrbld). **E**, **F** Release of IL-6 and IL-8 in LPS (100 ng/ml; 24 h)-stimulated cells. Knockdown of ANO6 (siANO6) strongly inhibits the release of IL-6 and IL-8. Mean ± SEM (number of experiments). Number sign (#) indicates significant inhibition of cytokine release by knockdown of ANO1 (*p* < 0.05; unpaired *t* test). Section sign (§) indicates significant increase of cytokine release by LPS (*p* < 0.05; unpaired *t* test)

We examined whether the release of IL-6 and IL-8 by differentiated BCi-NS1.1 human airway epithelial cells depends on the expression of ANO1 and ANO6 [20, 39]. In fact, basal and LPS-stimulated release of both cytokines were inhibited by siRNA-knockdown of ANO1 when compared to cells treated with scrambled RNA (scrbld) (Fig. 5A–C). Thus, these data suggest a proinflammatory role for ANO1 by supporting the release of cytokines, probably due to the pronounced impact of ANO1 on intracellular Ca^2+^ signalling, which determines exocytic activity (Fig. 10). Inhibition of IL-6/IL-8 release was even more pronounced by knockdown of ANO6, which is essential for exocytosis [10] (Fig. 5D–F). Thus, the present and previous data [7, 20, 67] suggested a role of both ANO1 and ANO6 for exocytosis of cytokines and mucins. While phospholipid scrambling by ANO6 is essential for exocytosis and membrane shedding [10, 30, 73], increase in intracellular sub-membranous Ca^2+^ by ANO1 supports the scrambling activity of ANO6 and supports activation of the exocytic SNARE complex [11, 37, 67] (Fig. 10).

### Niclosamide but not ivermectin acidifies cell pH and increases intracellular Ca^2+^ in BCi-NS1.1 cells

**Fig. 6:**
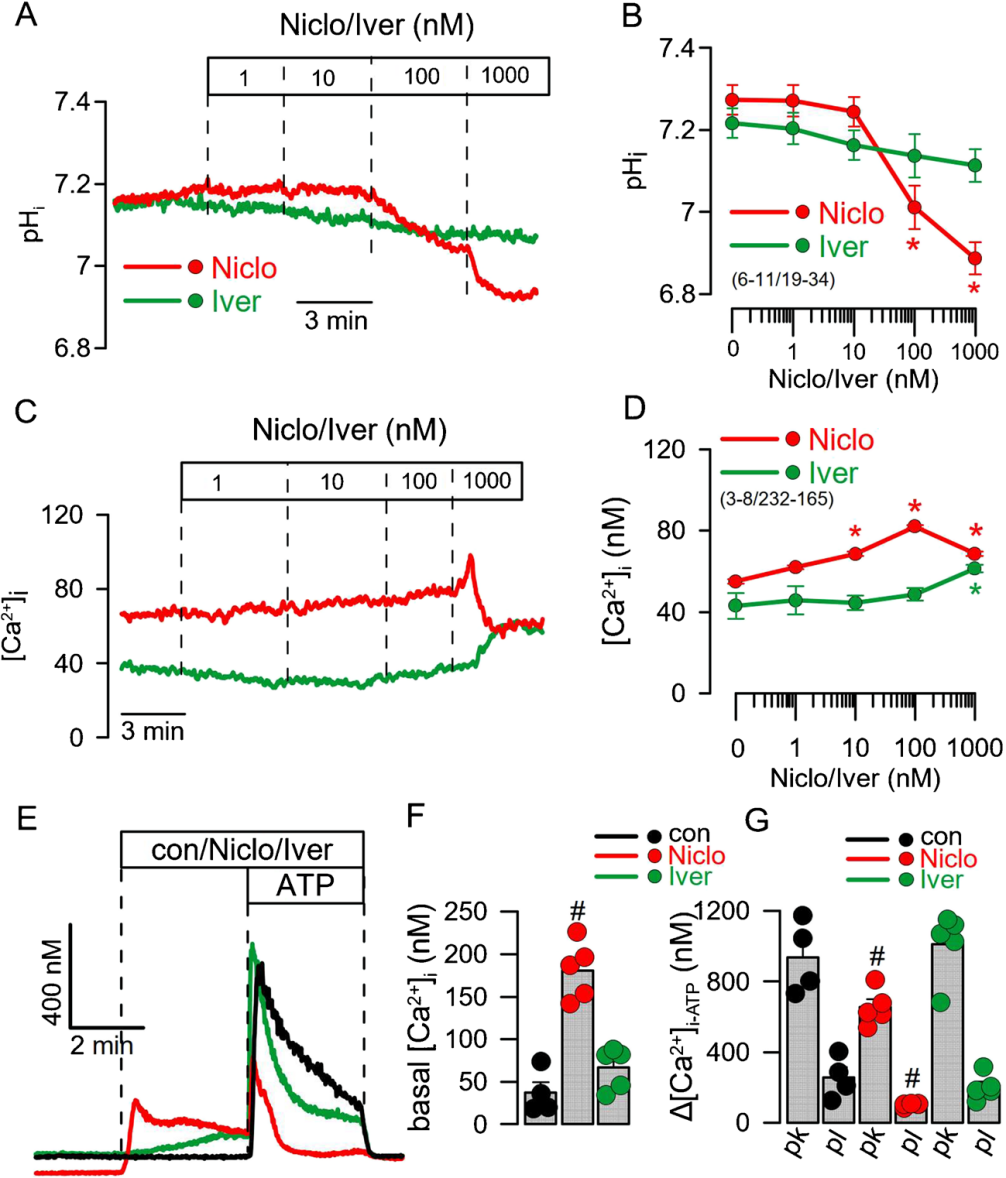
Effects of niclosamide and ivermectin on cytosolic pH and Ca^2+^ in BCi-NS1.1 cells. **A** Original tracings of intracellular pH assessed by BCECF, and effects of Niclo and Iver. **B** Concentration-dependent effects of Niclo and Iver. **C** Original tracings of intracellular Ca^2+^ measured by Fura-2 and effects of Niclo and Iver. **D** Concentration-dependent effects of Niclo and Iver. **E** Original tracings of intracellular Ca^2+^ and changes of [Ca^2+^]_*i*_ induced by ATP (100 µM) in the absence or presence of Niclo or Iver, respectively. **F**, **G** Effect of Niclo and Iver on basal Ca^2+^ and ATP-induced peak (pk) and plateau (pl) Ca^2+^-increase. Mean ± SEM (number of experiments). Asterisk (*) indicates significant increase or decrease, respectively (*p* < 0.05; paired *t* test). Number sign (#) indicates significant difference compared to con (*p* < 0.05; ANOVA)

Niclosamide was reported to alkalinize endosomal pH (thereby acidifying cytosolic pH) which could be a central mechanism for its antiviral effect [33, 41]. We examined whether therapeutic concentrations of Niclo and Iver also acidify cytosolic pH in airway epithelial cells. Human BCi-NS1.1 cells were loaded with the pH sensitive dye BCECF. Intracellular pH (pHi) was measured in the presence of increasing concentrations of Niclo or Iver. Cytosolic pH started to acidify when Niclo was applied at concentrations of 100 nM and higher (Fig. 6A, B). In contrast, Iver showed no effects on intracellular pH. When compared to the acidification induced by Niclo, intracellular Ca^2+^ ([Ca^2+^]_*i*_) was steadily enhanced by Niclo up to a concentration of 100 nM, while higher concentrations (up to 1 µM) induced a sudden Ca^2+^ increase (probably by Ca^2+^ release from the ER), with a subsequent drop in [Ca^2+^]_*i*_ [19, 55] (Fig. 6C, D). Iver had no effects on [Ca^2+^]_*i*_ at lower concentrations, but slightly increases [Ca^2+^]_*i*_ at 1 µM. As a result of ER Ca^2+^ store depletion by 1 µM Niclo [19], acute release of Ca^2+^ from the store by purinergic (ATP) stimulation was strongly inhibited. Again, Iver showed no effects on ATP-induced Ca^2+^ release (Fig. 6E–G). The present data indicate that at therapeutic concentrations detected in the plasma of treated patients, Niclo acidifies pHi and compromises Ca^2+^ signalling in differentiated airway epithelial cells.

### Niclosamide but not ivermectin inhibit ANO1 and ANO6

**Fig. 7:**
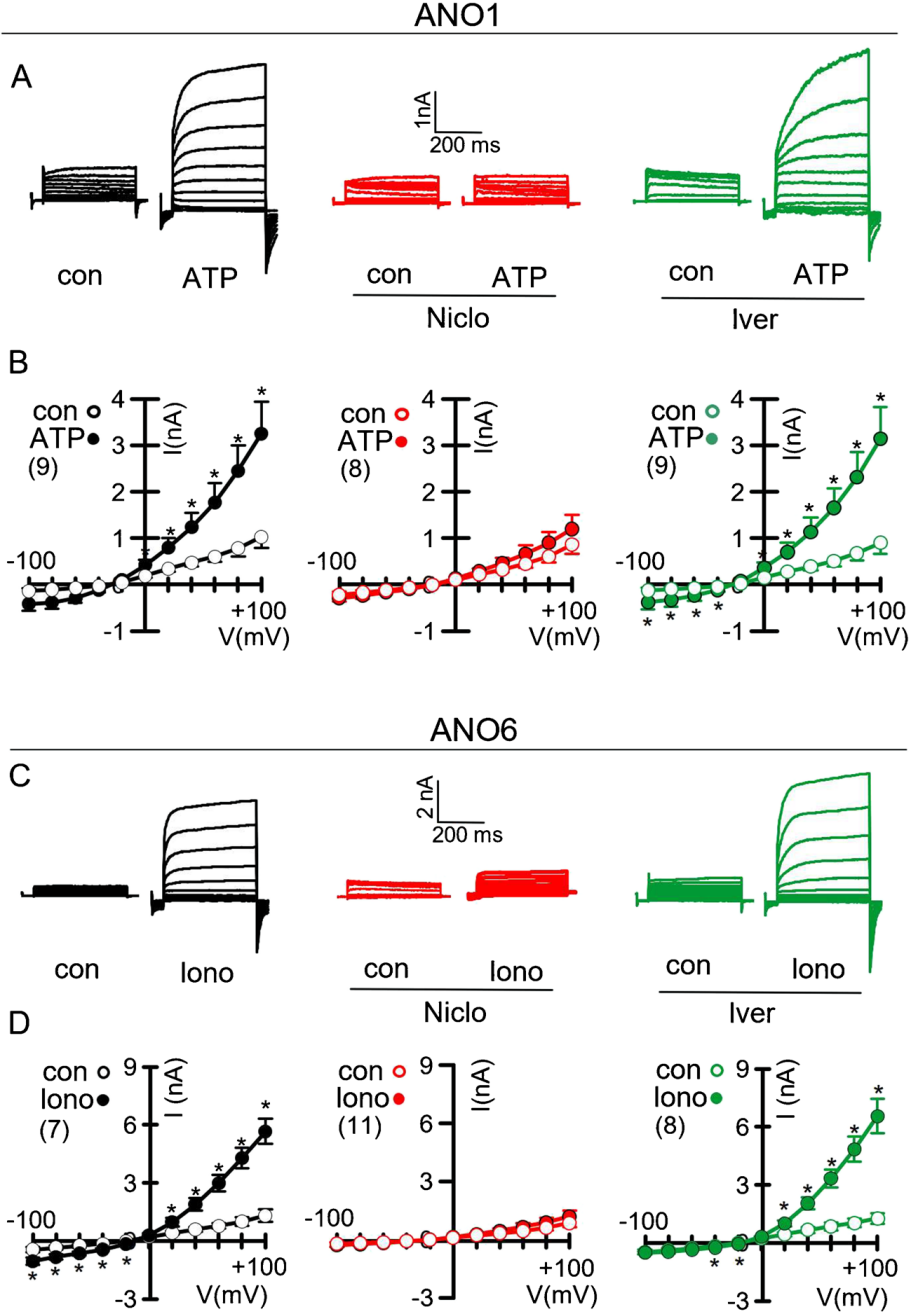
Niclosamide but not ivermectin inhibits activation of ANO1 or ANO6. **A** Whole-cell current overlays (clamp voltages ± 100 mV) indicate activation of ANO1 by ATP (100 µM) in overexpressing HEK293 cells, which is blocked by Niclo (1 µM) but not Iver (1 µM). **B** Corresponding current/voltage (I/V) relationships. **C**, **D** Whole-cell current overlays and I/V curves of ANO6 activated by ionomycin (1 µM) and inhibition by Niclo (1 µM) but not Iver (1 µM). Mock-transfected HEK293 cells did not activate a whole-cell current when stimulated by either ionomycin or ATP, and Niclo or Iver had no effects on basal whole-cell currents (not shown). Mean ± SEM (number of experiments). Asterisk (*) indicates significant activation by ATP or ionomycin (*p* < 0.05; paired *t* test)

The present data demonstrate cellular effects of Niclo, which cannot be reproduced by Iver at concentrations up to 1 µM. Inhibition of cytokine release by niclosamide may be due to the inhibition of the exocytic function of ANO1 and ANO6 [10, 20]. Here, we demonstrate that 1 µM Niclo completely inhibits whole cell currents activated by ATP or ionomycin in HEK293 cells overexpressing ANO1 and ANO6, respectively. Lower concentrations of Niclo at ~ 100–200 nM were shown earlier to inhibit anoctamins partially [12, 55]. Inhibition of anoctamins occurs directly (via binding to the hydrophobic binding pocket) and indirectly by lowering of intracellular Ca^2+^ concentrations [19, 35]. It should be noted that stimulation with ATP rises intracellular Ca^2+^ to levels sufficient for the activation of ANO1, but not of ANO6, which requires much higher Ca^2+^ concentrations. Therefore ANO6-expressing cells were stimulated by ionomycin instead of ATP. In contrast to Niclo, Iver did not inhibit the activation of the two anoctamins. It is therefore unlikely that the anti-inflammatory effects of Iver described earlier [29] are related to the inhibition of ANO1 or ANO6 (Fig. 7A, B).

### Niclosamide inhibits cytokine release at therapeutic concentrations but could be cytotoxic at concentrations above 500 nM

**Fig. 8:**
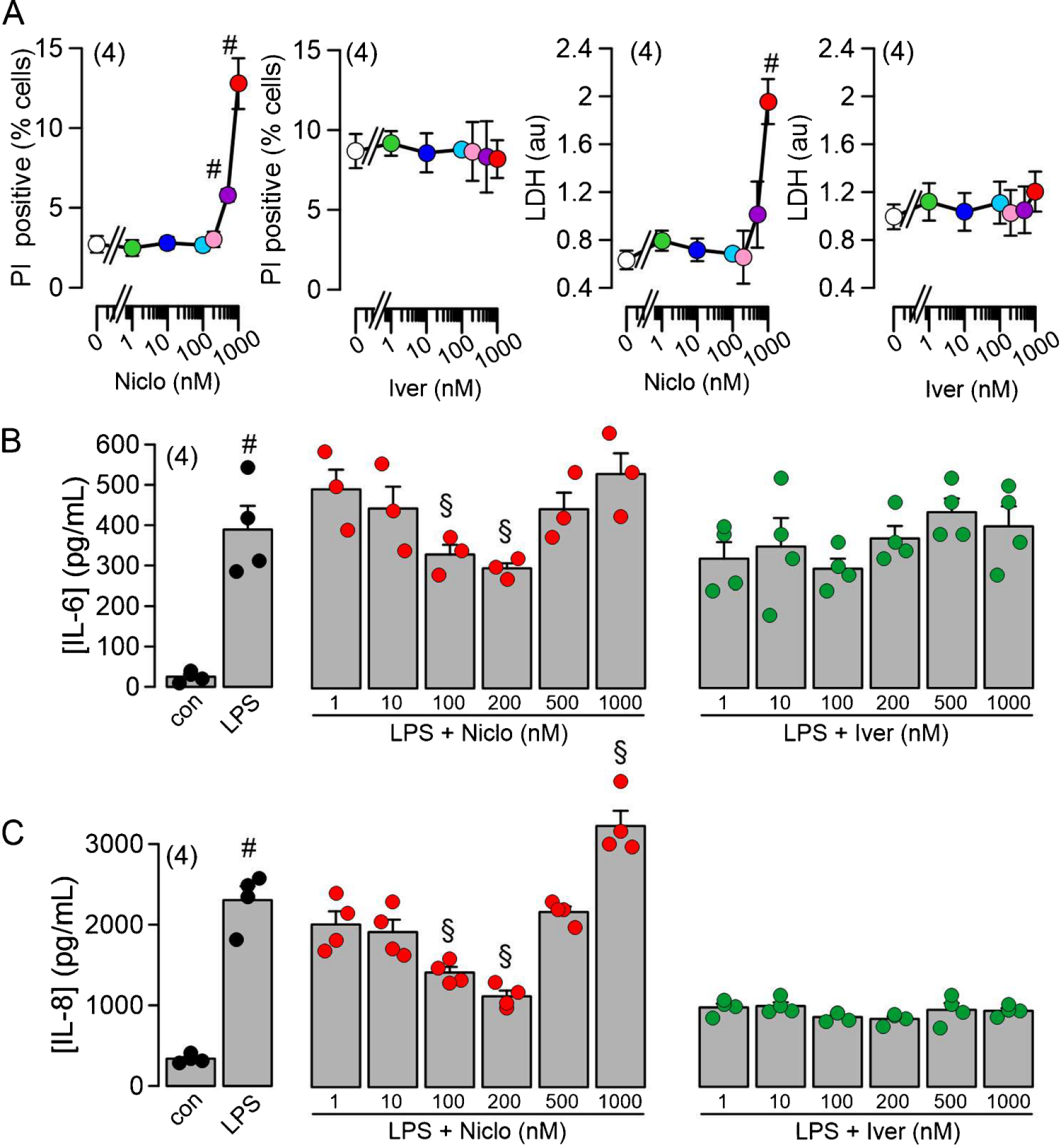
Niclosamide may exert cytotoxic effects on BCi-NS1.1 cells at concentrations ≥ 500 nM. **A** Effects of Niclo and Iver on propidium iodide (PI) uptake and LDH release. Niclo augmented PI positivity and LDH release at concentrations ≥ 500 nM. **B**, **C** Effects of different concentrations of Niclo and Iver on the release of IL-6 and IL-8, respectively. At ≥ 500 nM, Niclo no longer suppressed cytokine release, which may be due to a significant increase in intracellular Ca.^2+^. Mean ± SEM (number of assays). Number sign (#) indicates significant difference compared to 0 nM and con, respectively (*p* < 0.05; ANOVA). Section sign (§) indicates significant difference compared to LPS (*p* < 0.05; ANOVA)

When applied locally to mouse or human airways, very high concentrations of niclosamide (30 µM) did not exert any obvious toxic effects but reduced inflammatory symptoms (Fig. 1) [4]. We also examined how BCi-NS1.1 airway epithelial cells respond when exposed to concentrations of Niclo or Iver higher than 200 nM. To that end, the cells were incubated for 24 h at increasing concentrations of both drugs up to a maximal concentration of 1 µM. We found that at concentrations ≥ 500 nM, Niclo caused propidium iodide uptake and LDH-release. Moreover, the release of cytokines was inhibited at therapeutic concentrations of 100–200 nM, but the release was not inhibited at higher concentrations. These results are explained by the fact that Niclo transiently increases cytosolic [Ca^2+^] at concentrations of ≥ 500 nM, which further stimulates cytokine release. Again, Iver did not show any effects (Fig. 8A–C).

### Only high concentrations of niclosamide inhibit cytokine release from immune cells

**Fig. 9:**
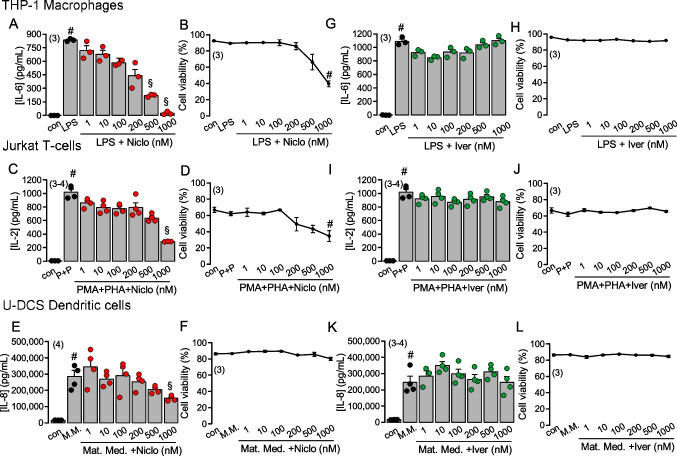
Inhibition of cytokine release by niclosamide but not ivermectin*.* **A**–**L** Concentration-dependent effects of Niclo and Iver on the release of cytokines and on cell viability of THP-1 macrophages, Jurkat T-cells, and U-DCS dendritic cells. Cytokine release was induced by LPS (24 h) in THP-1 cells (IL-6), by PMA/PHA (see “Materials and methods”; 24 h) in Jurkat cells (IL-2), and by maturation medium (Mat.Med.; see “Materials and methods”; 24 h) in U-DCS cells. **A**–**F** Only higher concentrations of Niclo above the typical therapeutic plasma concentrations inhibited cytokine release, or compromised cell viability. **G**–**L** Iver had no effects on cytokine release and did not show cytotoxic effects, even at concentrations far beyond therapeutic plasma concentrations. Mean ± SEM (number of assays). Number sign (#) indicates significant increase by LPS, PMA/PHA and Mat.Med. Section sign (§) indicates significant difference to LPS, PMA/PHA, and Mat.Med. (*p* < 0.05; unpaired *t* test)

In the present study, we show that Niclo (but not Iver) inhibits cytokine release by airway epithelial cells through inhibition of ANO1 and ANO6, and lowering cytoplasmic Ca^2+^ concentrations, which is likely to suppress lung inflammation. However, as these drugs may also inhibit the release of cytokines by immunologically active cells, we examined the effects of Niclo and Iver in THP-1 macrophages [24, 79], Jurkat T-cells [2], and U-DCS dendritic cells [54]. These cells express high levels of ANO6 but no ANO1 (https://www.proteinatlas.org (Fig. S3). Release of IL-6 by THP-1 macrophages was induced by LPS (24 h), release of IL-2 by Jurkat T-cells was induced by PMA/PHA (24 h), and release of IL-8 by U-DCS dendritic cells was induced by maturation medium (Mat.Med.; 24 h, see “Materials and methods”) (Fig. 9). Niclo (24 h) inhibited IL-6 release from THP-1 macrophages at concentrations ≥ 500 nM, while cytokine release by Jurkat T-cells and U-DCS dendritic cells was inhibited only at 1 µM niclosamide. Cell viability was only affected at high (1 µM) concentrations of Niclo (Fig. 9). Cell numbers were assessed before and after measurements in order to detect a possible loss of cells during the assay. Ivermectin did neither inhibit cytokine release nor decrease viability.

In conclusion, Niclo and Iver did not show toxic effects when applied directly *in vivo* to mouse airways at a high local concentration (30 µM). Airway mucus, the number of eosinophils and apoptotic cells were attenuated by Niclo but not by Iver. Cytokine release by airway epithelial cells *in vitro* was inhibited by Niclo at therapeutic concentrations of 100–200 nM, which is typically achieved as plasma concentrations during oral application. Higher concentrations of Niclo showed cytotoxic effect on cultured airway epithelial cells. Cytokine release by immune cells was inhibited only at ≥ 500 nM, and cell viability was affected only at the highest concentration of 1 µM. These results support the use of niclosamide as an anti-inflammatory drug in airways of patients with asthma, cystic fibrosis and COVID-19.

## Discussion

### Niclosamide but not ivermectin inhibits ANO1 and ANO6 and attenuates airway inflammation

**Fig. 10:**
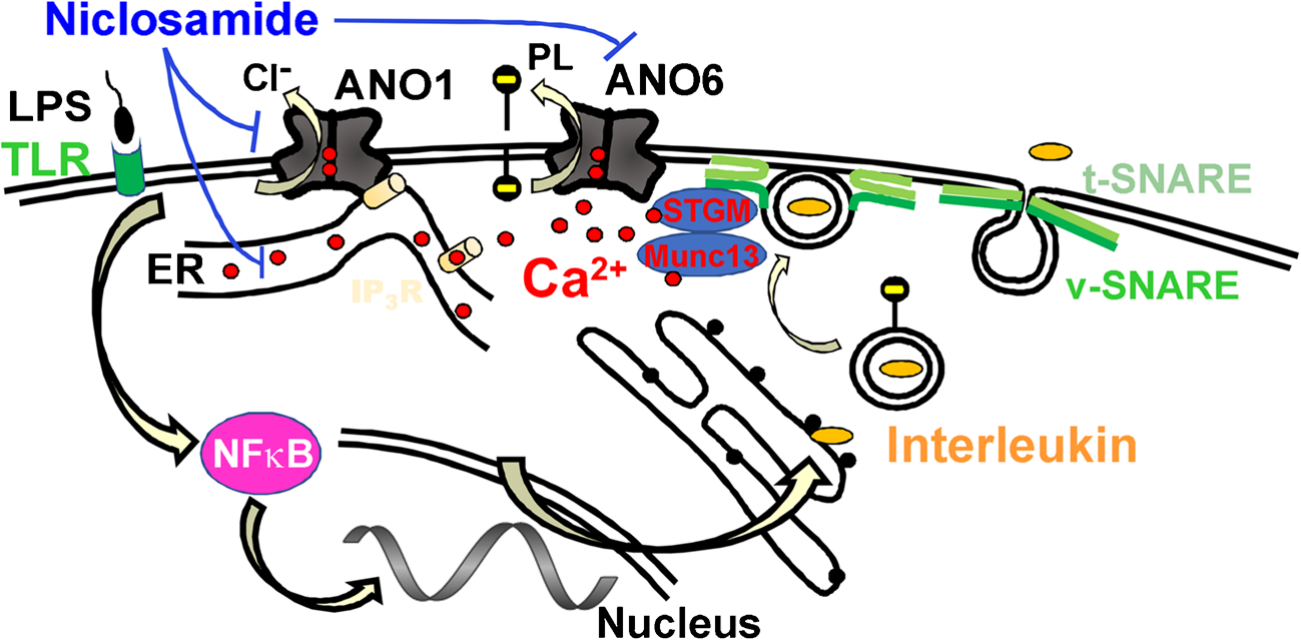
Proposed concept how ANO1 and ANO6 support cytokine release, which is inhibited by niclosamide. Toll-like receptors (TLR) sense bacterial lipopolysaccharides (LPS) which activate transcription of interleukins via nuclear factor kappa-B (NFχB) [77]. Interleukins are constitutively secreted by epithelial cells using the vesicular/target SNARE (soluble-*N*-ethylmaleimide–sensitive-factor accessory-protein receptor) machinery including the Ca^2+^ sensor synaptotagmin (STGM) and the Ca^2+^-binding protein Munc13 [74, 75]. ANO1 tethers the endoplasmic reticulum (ER) near the plasma membrane via binding to the Inositol trisphosphate receptor (IP_3_R), which enhances the sub-membranous Ca^2+^ concentration [11], thereby supporting Ca^2+^-dependent activation of synaptotagmin and phospholipid (PL) scrambling by ANO6 [58]. ANO1 was shown to interact within a network of SNARE, Munc and syntaxin proteins [60] and is regulated by extended synaptotagmin-1 (ESYT-1) [48]. Removal of negatively charged phospholipids (PL) from the inner plasma membrane leaflet supports vesicular fusion by lowering repulsive forces [30]. Niclosamide inhibits ANO1 and reduces ANO1 expression [15]. Niclosamide also directly lowers ER-Ca^2+^ content by inhibiting the sarcoplasmic endoplasmic reticulum Ca^2+^-ATPase (SERCA) and store-operated Ca^2+^ entry (SOCE) by inhibiting Orai channels [19], and inhibits PL scrambling by ANO6

The present data confirm our previous findings showing inhibition of ANO1 and ANO6 by niclosamide, and clearly indicate suppression of inflammatory symptoms. It has been argued that niclosamide directly reduces intracellular Ca^2+^ and thereby only indirectly inhibits ANO1 [28, 35]. However, in several previous studies, we and others demonstrated that (i) anoctamins, especially ANO1 supports Ca^2+^ release from the ER as well as reloading of the Ca^2+^ store by store operated Ca^2+^ entry (SOCE) [11, 38, 45], (ii) ANO1 knockout mice show strongly attenuated GPCR-induced Ca^2+^ signals in various tissues [7, 12, 69], (iii) niclosamide also inhibits ANO1, when intracellular Ca^2+^ is clamped to high concentrations [19, 55], (iv) in cells in which ANO1 was knocked out, niclosamide had no additional attenuating effect on intracellular Ca^2+^ signals [19], (v) the most specific ANO1 inhibitor Ani9 also inhibited GPCR-induced intracellular Ca^2+^ signals [19] and (vi) Ani9 had a similar inhibitory effect like niclosamide or benzbromarone, on renal cyst growth in polycystic kidneys *in vivo,* which is due to augmented Ca^2+^ signalling [15]. Further evidence for a direct role of ANO1 and ANO6 in exocytosis was provided in a recent report [20]. Figure 10 summarizes direct and circumstantial evidence for the role of ANO1 and ANO6 in exocytosis and cytokine release, and the effects of niclosamide.

### Appropriate therapeutic concentrations of niclosamide and potential toxicity

The use of high (≥ 1 µM) concentrations of niclosamide in cell culture experiments appears inappropriate and leads to the inhibition of the SERCA pump as well as Orai Ca^2+^ influx channels [19, 28, 35]. Artefactual inhibition of SERCA/Orai is often observed for amphipathic drugs and was also reported for the CFTR inhibitor CFTRinh-172, shown recently to compromise intracellular Ca^2+^ signalling [50]. When taken orally at the established and well-tolerated standard dose of 2 g/day niclosamide leads (in humans) to a variable peak plasma concentration of ~ 250 up to 6000 ng/ml (~ 700–18000 nM) [3, 72]. The effects of niclosamide *in vitro* described here were detected at concentrations ~ 10–50 times lower, which suggest oral dosages well below 2 g/day in order to dampen pulmonary inflammation. Analogues of niclosamide are currently developed with improved antiviral efficacy, lower cytotoxicity and improved pharmacokinetics [49]. Delivery to airways of nebulized niclosamide is currently explored and was found to be well tolerated in clinical trials. Local delivery of niclosamide allows higher concentrations in the airway surface liquid, improves pharmacokinetic profiles and lowers systemic concentrations, thereby reducing toxicity [4, 57, 81]. The present data show positive effects of niclosamide and no toxicity even when applied at high concentrations to asthmatic mouse airways (Fig. 1). In contrast to niclosamide, ivermectin (maximal therapeutic plasma concentration ~ 50 nM [66]) did neither inhibit anoctamins nor reduce cytokine release even at 1 µM.

### Pleotropic effects of niclosamide

Inflamed airways show high levels of IL-13 (asthma [53, 55]), IL-8 (CF, [6, 40]) or IL-6 (COVID-19; cytokine storm, [32]). Niclosamide may be useful in lowering airway cytokine levels without compromising local immune defence. Moreover, enhancing endosomal pH and lowering intracellular Ca^2+^ levels by niclosamide may both reduce viral uptake [23, 25, 84]. Finally, niclosamide inhibits the formation of pneumocyte syncytia and thrombus formation induced by the spike protein of SARS-CoV-2, which is due to the inhibition of ANO6 and possibly ANO1 [5, 9, 18, 71]. Taken together, these findings support further evaluation of niclosamide for the use in inflammatory airway diseases.

### Supplementary Information

Below is the link to the electronic supplementary material.

Supplementary file1 (PDF 55 KB)

Supplementary file2 (PDF 50 KB)

Supplementary file3 (PDF 251 KB)

Supplementary file4 (PDF 1410 KB)

## Data Availability

Original data are available on request.

## References

[1] Abd-Elsalam S, Noor RA, Badawi R, Khalaf M, Esmail ES, Soliman S, Abd El Ghafar MS, Elbahnasawy M, Moustafa EF, Hassany SM, Medhat MA, Ramadan HK, Eldeen MAS, Alboraie M, Cordie A, Esmat G (2021). Clinical study evaluating the efficacy of ivermectin in COVID-19 treatment: a randomized controlled study. J Med Virol.

[2] Abraham RT, Weiss A (2004). Jurkat T cells and development of the T-cell receptor signalling paradigm. Nat Rev Immunol.

[3] Andrews P, Thyssen J, Lorke D (1982). The biology and toxicology of molluscicides, Bayluscide. Pharmacol Ther.

[4] Backer V, Sjöbring U, Sonne J, Weiss A, Hostrup M, Johansen HK, Becker V, Sonne DP, Balchen T, Jellingsø M, Sommer MOA (2021). A randomized, double-blind, placebo-controlled phase 1 trial of inhaled and intranasal niclosamide: a broad spectrum antiviral candidate for treatment of COVID-19. Lancet Reg Health Eur.

[5] Baig AA, Haining EJ, Geuss E, Beck S, Swieringa F, Wanitchakool P, Schuhmann MK, Stegner D, Kunzelmann K, Kleinschnitz C, Heemskerk JW, Braun A, Nieswandt B (2016). TMEM16F-mediated platelet membrane phospholipid scrambling is critical for hemostasis and thrombosis but not thromboinflammation in mice. Arterioscler Thromb Vasc Biol.

[6] Bautista MV, Chen Y, Ivanova VS, Rahimi MK, Watson AM, Rose MC (2009). IL-8 regulates mucin gene expression at the posttranscriptional level in lung epithelial cells. J Immunol.

[7] Benedetto R, Cabrita I, Schreiber R, Kunzelmann K (2019). TMEM16A is indispensable for basal mucus secretion in airways and intestine. FASEB J.

[8] Benedetto R, Centeio R, Ousingsawat J, Schreiber R, Janda M, Kunzelmann K (2020). Transport properties in CFTR-/- knockout piglets suggest normal airway surface liquid pH and enhanced amiloride-sensitive Na(+) absorption. Pflugers Arch.

[9] Braga L, Ali H, Secco I, Chiavacci E, Neves G, Goldhill D, Penn R, Jimenez-Guardeño JM, Ortega-Prieto AM, Bussani R, Cannatà A, Rizzari G, Collesi C, Schneider E, Arosio D, Shah AM, Barclay WS, Malim MH, Burrone J, Giacca M (2021). Drugs that inhibit TMEM16 proteins block SARS-CoV-2 spike-induced syncytia. Nature.

[10] Bricogne C, Fine M, Pereira PM, Sung J, Tijani M, Wang Y, Henriques R, Collins MK, Hilgemann D (2019). TMEM16F activation by Ca(2+) triggers plasma membrane expansion and directs PD-1 trafficking. Sci Rep.

[11] Cabrita I, Benedetto R, Fonseca A, Wanitchakool P, Sirianant L, Skryabin BV, Schenk LK, Pavenstadt H, Schreiber R, Kunzelmann K (2017). Differential effects of anoctamins on intracellular calcium signals. Faseb j.

[12] Cabrita I, Benedetto R, Schreiber R, Kunzelmann K (2019). Niclosamide repurposed for the treatment of inflammatory airway disease. JCI insight.

[13] Cabrita I, Benedetto R, Wanitchakool P, Lerias J, Centeio R, Ousingsawat J, Schreiber R, Kunzelmann K (2020). TMEM16A mediated mucus production in human airway epithelial cells. Am J Respir Cell Mol Biol.

[14] Cabrita I, Buchholz B, Schreiber R, Kunzelmann K (2020). TMEM16A drives renal cyst growth by augmenting Ca(2+) signaling in M1 cells. J Mol Med (Berl).

[15] Cabrita I, Kraus A, Scholz JK, Skoczynski K, Schreiber R, Kunzelmann K, Buchholz B (2020). Cyst growth in ADPKD is prevented by pharmacological and genetic inhibition of TMEM16A in vivo. Nat Commun.

[16] Cabrita I, Talbi K, Kunzelmann K, Schreiber R (2021). Loss of PKD1 and PKD2 share common effects on intracellular Ca2+ signaling. Cell Calcium.

[17] Caly L, Druce JD, Catton MG, Jans DA, Wagstaff KM (2020). The FDA-approved drug ivermectin inhibits the replication of SARS-CoV-2 in vitro. Antiviral Res.

[18] Cappelletto A, Allan HE, Crescente M, Schneider E, Bussani R, Ali H, Secco I, Vodret S, Simeone R, Mascaretti L, Zacchigna S, Warner TD, Giacca M (2022). SARS-CoV-2 Spike protein activates TMEM16F-mediated platelet procoagulant activity. Front Cardiovasc Med.

[19] Centeio R, Cabrita I, Benedetto R, Talbi K, Ousingsawat J, Schreiber R, Sullivan JK, Kunzelmann K (2020). Pharmacological inhibition and activation of the Ca(2+) activated Cl(-) channel TMEM16A. Int J Mol Sci.

[20] Centeio R, Cabrita I, Schreiber R, Kunzelmann K (2023). TMEM16A/F support exocytosis but do not inhibit Notch-mediated goblet cell metaplasia of BCi-NS1.1 human airway epithelium. Front Physiol.

[21] Centeio R, Ousingsawat J, Cabrita I, Schreiber R, Talbi K, Benedetto R, Doušová T, Verbeken EK, De Boeck K, Cohen I, Kunzelmann K (2021) Mucus release and airway constriction by TMEM16A may worsen pathology in inflammatory lung disease. Int J Mol Sci 22. 10.3390/ijms22157852 10.3390/ijms22157852 PMC8346050 34360618

[22] Centeio R, Ousingsawat J, Schreiber R, Kunzelmann K (2021). CLCA1 regulates airway mucus production and ion secretion through TMEM16A. Int J Mol Sci.

[23] Chang-Graham AL, Perry JL, Strtak AC, Ramachandran NK, Criglar JM, Philip AA, Patton JT, Estes MK, Hyser JM (2019). Rotavirus calcium dysregulation manifests as dynamic calcium signaling in the cytoplasm and endoplasmic reticulum. Sci Rep.

[24] Chanput W, Mes JJ, Wichers HJ (2014). THP-1 cell line: an in vitro cell model for immune modulation approach. Int Immunopharmacol.

[25] Chen X, Cao R, Zhong W (2019). Host calcium channels and pumps in viral infections. Cells.

[26] Ching JC, Lobanova L, Loewen ME (2013). Secreted hCLCA1 is a signaling molecule that activates airway macrophages. PLoS One.

[27] Choi HI, Kim T, Lee SW, Woo Kim J, Ju Noh Y, Kim GY, Jin Park H, Chae YJ, Lee KR, Kim SJ, Koo TS (2021). Bioanalysis of niclosamide in plasma using liquid chromatography-tandem mass and application to pharmacokinetics in rats and dogs. J Chromatogr, B: Anal Technol Biomed Life Sci.

[28] Danahay H, Lilley S, Adley K, Charlton H, Fox R, Gosling M (2023). Niclosamide does not modulate airway epithelial function through blocking of the calcium activated chloride channel, TMEM16A. Front Pharmacol.

[29] de Melo GD, Lazarini F, Larrous F, Feige L, Kornobis E, Levallois S, Marchio A, Kergoat L, Hardy D, Cokelaer T, Pineau P, Lecuit M, Lledo PM, Changeux JP, Bourhy H (2021). Attenuation of clinical and immunological outcomes during SARS-CoV-2 infection by ivermectin. EMBO Mol Med.

[30] Deisl C, Hilgemann DW, Syeda R, Fine M (2021). TMEM16F and dynamins control expansive plasma membrane reservoirs. Nat Commun.

[31] Dietert K, Reppe K, Mundhenk L, Witzenrath M, Gruber AD (2014). mCLCA3 modulates IL-17 and CXCL-1 induction and leukocyte recruitment in murine Staphylococcus aureus pneumonia. PLoS One.

[32] Fajgenbaum DC, June CH (2020). Cytokine storm. N Engl J Med.

[33] Fonseca BD, Diering GH, Bidinosti MA, Dalal K, Alain T, Balgi AD, Forestieri R, Nodwell M, Rajadurai CV, Gunaratnam C, Tee AR, Duong F, Andersen RJ, Orlowski J, Numata M, Sonenberg N, Roberge M (2012). Structure-activity analysis of niclosamide reveals potential role for cytoplasmic pH in control of mammalian target of rapamycin complex 1 (mTORC1) signaling. J Biol Chem.

[34] Gassen NC, Papies J, Bajaj T, Emanuel J, Dethloff F, Chua RL, Trimpert J, Heinemann N, Niemeyer C, Weege F, Hönzke K, Aschman T, Heinz DE, Weckmann K, Ebert T, Zellner A, Lennarz M, Wyler E, Schroeder S, Richter A, Niemeyer D, Hoffmann K, Meyer TF, Heppner FL, Corman VM, Landthaler M, Hocke AC, Morkel M, Osterrieder N, Conrad C, Eils R, Radbruch H, Giavalisco P, Drosten C, Müller MA (2021). SARS-CoV-2-mediated dysregulation of metabolism and autophagy uncovers host-targeting antivirals. Nat Commun.

[35] Genovese M, Buccirossi M, Guidone D, De Cegli R, Sarnataro S, di Bernardo D, Galietta LJV (2022). Analysis of a panel of tmem16a chloride channel inhibitors reveals indirect mechanisms involving alteration of calcium signaling. Br J Pharmacol.

[36] Grynkiewicz G, Poenie M, Tsien RY (1985). A new generation of Ca^2+^ indicators with greatly improved fluorescence properties. J Biol Chem.

[37] Henkel B, Drose DR, Ackels T, Oberland S, Spehr M, Neuhaus EM (2015). Co-expression of anoctamins in cilia of olfactory sensory neurons. Chem Senses.

[38] Jin X, Shah S, Liu Y, Zhang H, Lees M, Fu Z, Lippiat JD, Beech DJ, Sivaprasadarao A, Baldwin SA, Zhang H, Gamper N (2013). Activation of the Cl- channel ANO1 by localized calcium signals in nociceptive sensory neurons requires coupling with the IP3 receptor. Sci Signal.

[39] Jo S, Centeio R, Park J, Ousingsawat J, Jeon DK, Talbi K, Schreiber R, Ryu K, Kahlenberg K, Somoza V, Delpiano L, Gray MA, Amaral MD, Railean V, Beekman JM, Rodenburg LW, Namkung W, Kunzelmann K (2022). The SLC26A9 inhibitor S9–A13 provides no evidence for a role of SLC26A9 in airway chloride secretion but suggests a contribution to regulation of ASL pH and gastric proton secretion. Faseb J.

[40] Jundi K, Greene CM (2015). Transcription of interleukin-8: how altered regulation can affect cystic fibrosis lung disease. Biomolecules.

[41] Jurgeit A, McDowell R, Moese S, Meldrum E, Schwendener R, Greber UF (2012). Niclosamide is a proton carrier and targets acidic endosomes with broad antiviral effects. PLoS Pathog.

[42] Keeler SP, Yantis J, Gerovac BJ, Youkilis SL, Podgorny S, Mao D, Zhang Y, Whitworth KM, Redel B, Samuel MS, Wells KD, Prather RS, Holtzman MJ (2022). Chloride channel accessory 1 gene deficiency causes selective loss of mucus production in a new pig model. Am J Physiol Lung Cell Mol Physiol.

[43] Kodama T, Matsuyama T, Miyata S, Nishimura H, Nishioka Y, Kitada O, Sugita M (1998). Kinetics of apoptosis in the lung of mice with allergic airway inflammation. Clin Exp Allergy.

[44] Kunzelmann K, Ousingsawat J, Benedetto R, Cabrita I, Schreiber R (2019). Contribution of anoctamins to cell survival and cell death. Cancers.

[45] Kunzelmann K, Ousingsawat J, Cabrita I, Doušová T, Bähr A, Janda M, Schreiber R, Benedetto R (2019). TMEM16A in cystic fibrosis: activating or inhibiting?. Front Pharmacol.

[46] Laing R, Gillan V, Devaney E (2017). Ivermectin - old drug, new tricks?. Trends Parasitol.

[47] Lerias J, Pinto M, Benedetto R, Schreiber R, Amaral M, Aureli M, Kunzelmann K (2018). Compartmentalized crosstalk of CFTR and TMEM16A (ANO1) through EPAC1 and ADCY1. Cell Signal.

[48] Lerias JR, Pinto MC, Botelho HM, Awatade NT, Quaresma MC, Silva IAL, Wanitchakool P, Schreiber R, Pepperkok R, Kunzelmann K, Amaral MD (2018). A novel microscopy-based assay identifies extended synaptotagmin-1 (ESYT1) as a positive regulator of anoctamin 1 traffic. Biochim Biophys Acta.

[49] Li R, Zhang Z, Huang S, Peng K, Jiang H, Shen J, Zhang B, Jiang X (2023). Synthesis, cytotoxicity, and pharmacokinetic evaluations of niclosamide analogs for anti-SARS-CoV-2. Eur J Med Chem.

[50] Lin J, Gettings SM, Talbi K, Schreiber R, Taggart MJ, Preller M, Kunzelmann K, Althaus M, Gray MA (2023). Pharmacological inhibitors of the cystic fibrosis transmembrane conductance regulator exert off-target effects on epithelial cation channels. Pflugers Arch.

[51] Lott K, Cingolani G (2011). The importin β binding domain as a master regulator of nucleocytoplasmic transport. Biochim Biophys Acta.

[52] Lu S, Kolls JK (2023). Multi-omic comparisons between CFBE41o-cells stably expressing wild-type CFTR and F508del-mutant CFTR. J Cyst Fibros: official journal of the European Cystic Fibrosis Society.

[53] Marone G, Granata F, Pucino V, Pecoraro A, Heffler E, Loffredo S, Scadding GW, Varricchi G (2019). The intriguing role of interleukin 13 in the pathophysiology of asthma. Front Pharmacol.

[54] Mellert K, Benckendorff J, Leithäuser F, Zimmermann K, Wiegand P, Frascaroli G, Buck M, Malaise M, Hartmann G, Barchet W, Fürst D, Mytilineos J, Mayer-Steinacker R, Viardot A, Möller P (2020). U-DCS: characterization of the first permanent human dendritic sarcoma cell line. Sci Rep.

[55] Miner K, Labitzke K, Liu B, Elliot R, Wang P, Henckels K, Gaida K, Elliot R, Chen JJ, Liu L, Leith A, Trueblood E, Hensley K, Xia X-Z, Homann O, Bennett B, Fiorino M, Whoriskey J, Yu G, Escobar S, Wong M, Born TL, Budelsky A, Comeau M, Smith D, Phillips J, Johnston JA, McGivern JG, Weikl K, Powers D, Kunzelmann K, Mohn D, Hochheimer A, Sullivan JK (2019). Drug repurposing: the anthelmintics niclosamide and nitazoxanide are potent TMEM16A antagonists that fully bronchodilate airways. Front Pharmacol.

[56] Mundhenk L, Erickson NA, Klymiuk N, Gruber AD (2018). Interspecies diversity of chloride channel regulators, calcium-activated 3 genes. PLoS One.

[57] Ousingsawat J, Centeio R, Cabrita I, Talbi K, Zimmer O, Graf M, Göpferich A, Schreiber R, Kunzelmann K (2022). Airway delivery of hydrogel-encapsulated niclosamide for the treatment of inflammatory airway disease. Int J Mol Sci.

[58] Ousingsawat J, Schreiber R, Kunzelmann K (2019) TMEM16F/anoctamin 6 in Ferroptotic Cell Death. Cancers 11:pii: E625. 10.3390/cancers11030382 10.3390/cancers11050625 PMC6562394 31060306

[59] Patil VM, Verma S, Masand N (2022). Prospective mode of action of ivermectin: SARS-CoV-2. Eur J Med Chem Rep.

[60] Perez-Cornejo P, Gokhale A, Duran C, Cui Y, Xiao Q, Hartzell HC, Faundez V (2012). Anoctamin 1 (Tmem16A) Ca2+-activated chloride channel stoichiometrically interacts with an ezrin-radixin-moesin network. Proc Natl Acad Sci U S A.

[61] Portmann-Baracco A, Bryce-Alberti M, Accinelli RA (2020). Antiviral and anti-inflammatory properties of ivermectin and its potential use in COVID-19. Arch Bronconeumol.

[62] Prabhakara C, Godbole R, Sil P, Jahnavi S, Gulzar SE, van Zanten TS, Sheth D, Subhash N, Chandra A, Shivaraj A, Panikulam P, Ibrahim U, Nuthakki VK, Puthiyapurayil TP, Ahmed R, Najar AH, Lingamallu SM, Das S, Mahajan B, Vemula P, Bharate SB, Singh PP, Vishwakarma R, Guha A, Sundaramurthy V, Mayor S (2021). Strategies to target SARS-CoV-2 entry and infection using dual mechanisms of inhibition by acidification inhibitors. PLoS Pathog.

[63] Riley RS, Ben-Ezra JM, Massey D, Slyter RL, Romagnoli G (2004). Digital photography: a primer for pathologists. J Clin Lab Anal.

[64] Rüter A, Gunzer U (1984). Differentiation of granulocytes in Pappenheim stained blood cell smears using standardized cytophotometric analysis. Blut.

[65] Sala-Rabanal M, Yurtsever Z, Nichols CG, Brett TJ (2015). Secreted CLCA1 modulates TMEM16A to activate Ca(2+)-dependent chloride currents in human cells. Elife.

[66] Schmith VD, Zhou JJ, Lohmer LRL (2020). The approved dose of ivermectin alone is not the Ideal dose for the treatment of COVID-19. Clin Pharmacol Ther.

[67] Schreiber R, Cabrita I, Kunzelmann K (2022). Paneth cell secretion in vivo requires expression of Tmem16a and Tmem16f. Gastro Hep Adv.

[68] Schreiber R, Castrop H, Kunzelmann K (2008). Allergen induced airway hyperresponsiveness is absent in ecto-5´-nucleotidase (CD73) deficient mice. Pflugers Arch.

[69] Schreiber R, Faria D, Skryabin BV, Rock JR, Kunzelmann K (2015). Anoctamins support calcium-dependent chloride secretion by facilitating calcium signaling in adult mouse intestine. Pflügers Arch.

[70] Schreiber R, Uliyakina I, Kongsuphol P, Warth R, Mirza M, Martins JR, Kunzelmann K (2010). Expression and function of epithelial anoctamins. J Biol Chem.

[71] Sim JR, Shin DH, Park PG, Park SH, Bae JY, Lee Y, Kang DY, Kim YJ, Aum S, Noh SH, Hwang SJ, Cha HR, Kim CB, Ko SH, Park S, Jeon D, Cho S, Lee GE, Kim J, Moon YH, Kim JO, Nam JS, Kim CH, Moon S, Chung YW, Park MS, Ryu JH, Namkung W, Lee JM, Lee MG (2022). Amelioration of SARS-CoV-2 infection by ANO6 phospholipid scramblase inhibition. Cell Rep.

[72] Singh S, Weiss A, Goodman J, Fisk M, Kulkarni S, Lu I, Gray J, Smith R, Sommer M, Cheriyan J (2022). Niclosamide-A promising treatment for COVID-19. Br J Pharmacol.

[73] Sommer A, Kordowski F, Buch J, Maretzky T, Evers A, Andra J, Dusterhoft S, Michalek M, Lorenzen I, Somasundaram P, Tholey A, Sonnichsen FD, Kunzelmann K, Heinbockel L, Nehls C, Gutsmann T, Grotzinger J, Bhakdi S, Reiss K (2016). Phosphatidylserine exposure is required for ADAM17 sheddase function. Nat Commun.

[74] Stow JL, Manderson AP, Murray RZ (2006). SNAREing immunity: the role of SNAREs in the immune system. Nat Rev Immunol.

[75] Südhof TC, Rothman JE (2009). Membrane fusion: grappling with SNARE and SM proteins. Science.

[76] Svenningsen P, Nielsen MR, Marcussen N, Walter S, Jensen BL (2014). TMEM16A is a Ca(2+) -activated Cl(-) channel expressed in the renal collecting duct. Acta Physiol (Oxf).

[77] Tanaka T, Narazaki M, Kishimoto T (2014). IL-6 in inflammation, immunity, and disease. Cold Spring Harb Perspect Biol.

[78] Walters MS, Gomi K, Ashbridge B, Moore MA, Arbelaez V, Heldrich J, Ding BS, Rafii S, Staudt MR, Crystal RG (2013). Generation of a human airway epithelium derived basal cell line with multipotent differentiation capacity. Respir Res.

[79] Wanitchakool P, Ousingsawat J, Sirianant L, Cabrita I, Faria D, Schreiber R, Kunzelmann K (2017). Cellular defects by deletion of ANO10 are due to deregulated local calcium signaling. Cell Signal.

[80] Weinbach EC, Garbus J (1969). Mechanism of action of reagents that uncouple oxidative phosphorylation. Nature.

[81] Weiss A, Bischof RJ, Landersdorfer CB, Nguyen TH, Davies A, Ibrahim J, Wynne P, Wright P, Ditzinger G, Montgomery AB, Meeusen E, McIntosh MP, Sommer MO (2023). Single-dose pharmacokinetics and lung function of nebulized niclosamide ethanolamine in sheep. Pharm Res.

[82] Wu CJ, Jan JT, Chen CM, Hsieh HP, Hwang DR, Liu HW, Liu CY, Huang HW, Chen SC, Hong CF, Lin RK, Chao YS, Hsu JT (2004). Inhibition of severe acute respiratory syndrome coronavirus replication by niclosamide. Antimicrob Agents Chemother.

[83] Yan S, Ci X, Chen N, Chen C, Li X, Chu X, Li J, Deng X (2011). Anti-inflammatory effects of ivermectin in mouse model of allergic asthma. Inflamm Res: official journal of the European Histamine Research Society [et al].

[84] Yang Y, Yang P, Huang C, Wu Y, Zhou Z, Wang X, Wang S (2021). Inhibitory effect on SARS-CoV-2 infection of neferine by blocking Ca(2+)-dependent membrane fusion. J Med Virol.

