# Supplementary material for: Niclosamide, but not ivermectin, inhibits anoctamin 1 and 6 and attenuates inflammation of the respiratory tract
Source: Pflugers Arch. 2023 Nov 18;476(2):211–27. doi: 10.1007/s00424-023-02878-w (PMC10791962; doi:10.1007/s00424-023-02878-w)
Supplement: Supplementary file 1 — Supplementary file1 (PDF 55 KB) [file 424_2023_2878_MOESM1_ESM.pdf]

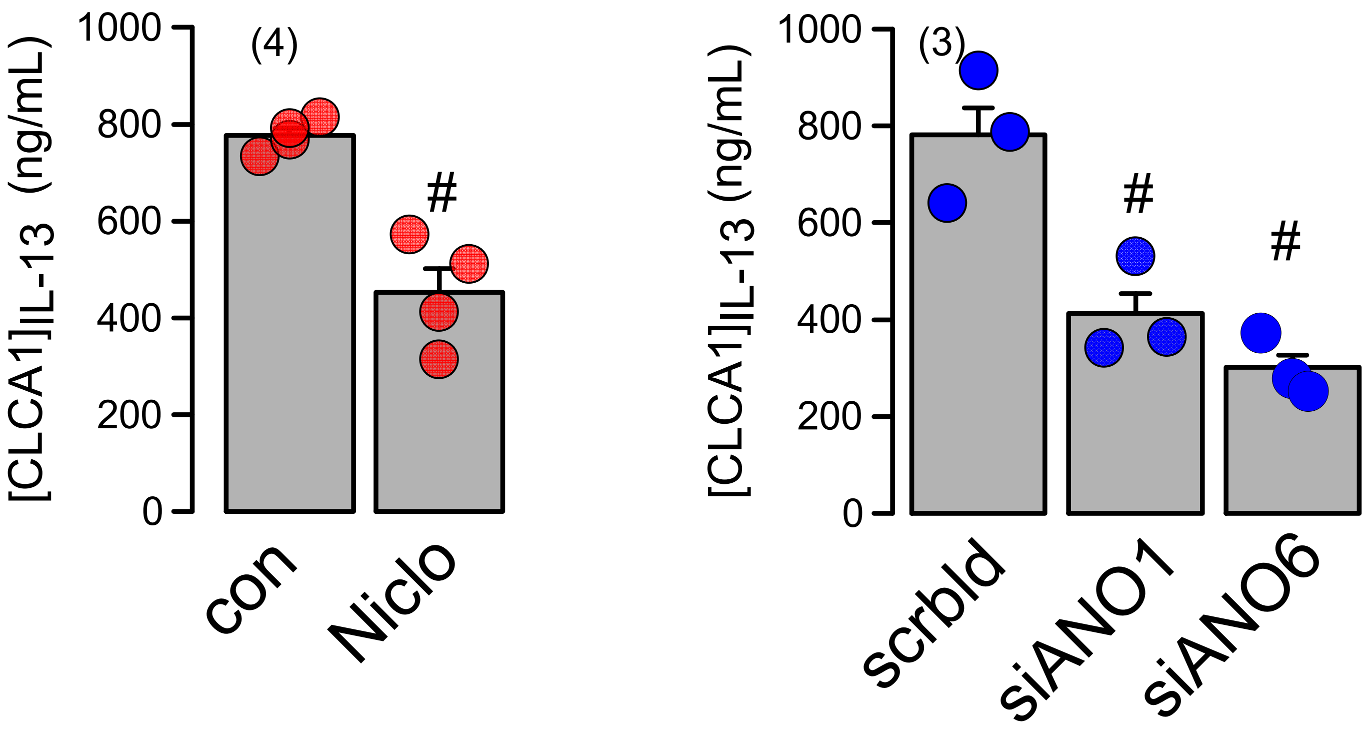

Supplementary Figure 1. *Inhibition of IL-13 - induced CLCA1 release by niclosamide and siRNA knockdown of ANO1 or ANO6.* Summary of CLCA1-release from CFBE human airway epithelial cells after incubation of the cells for 48 hrs with IL-13 (100 ng/ml). Cells were exposed to niclosamide (1 µM) or scrambled RNA, siRNA-ANO1 or siRNA-ANO6 for 72 hrs $^{\#}$($p<0.05$; unpaired t-test).
