# Supplementary material for: Niclosamide, but not ivermectin, inhibits anoctamin 1 and 6 and attenuates inflammation of the respiratory tract
Source: Pflugers Arch. 2023 Nov 18;476(2):211–27. doi: 10.1007/s00424-023-02878-w (PMC10791962; doi:10.1007/s00424-023-02878-w)
Supplement: Supplementary file 2 — Supplementary file2 (PDF 50 KB) [file 424_2023_2878_MOESM2_ESM.pdf]

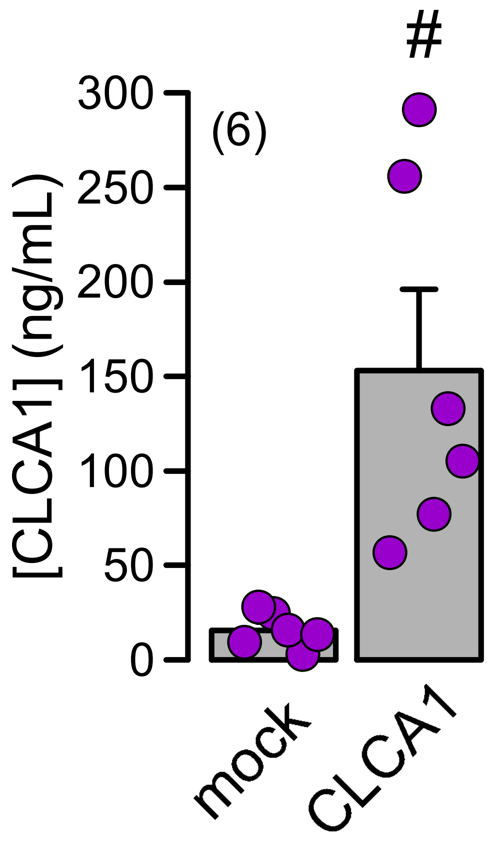


**Supplementary Figure 2**. *CLCA1-induced CLCA1 release.* Summary of CLCA1-release from BCi-NS1.1 human airway epithelial cells after preincubation of the cells for 24 hrs with supernatant from mock transfected HEK293 cells or cells overexpressing hCLCA1, which secrete cleaved (N-terminal) CLCA1 into the supernatant. Release of CLCA1 is augmented after preincubation with secreted CLCA1. #significant difference to mock ($p<0.05$; unpaired t-test).
