# Supplementary material for: Niclosamide, but not ivermectin, inhibits anoctamin 1 and 6 and attenuates inflammation of the respiratory tract
Source: Pflugers Arch. 2023 Nov 18;476(2):211–27. doi: 10.1007/s00424-023-02878-w (PMC10791962; doi:10.1007/s00424-023-02878-w)
Supplement: Supplementary file 3 — Supplementary file3 (PDF 251 KB) [file 424_2023_2878_MOESM3_ESM.pdf]

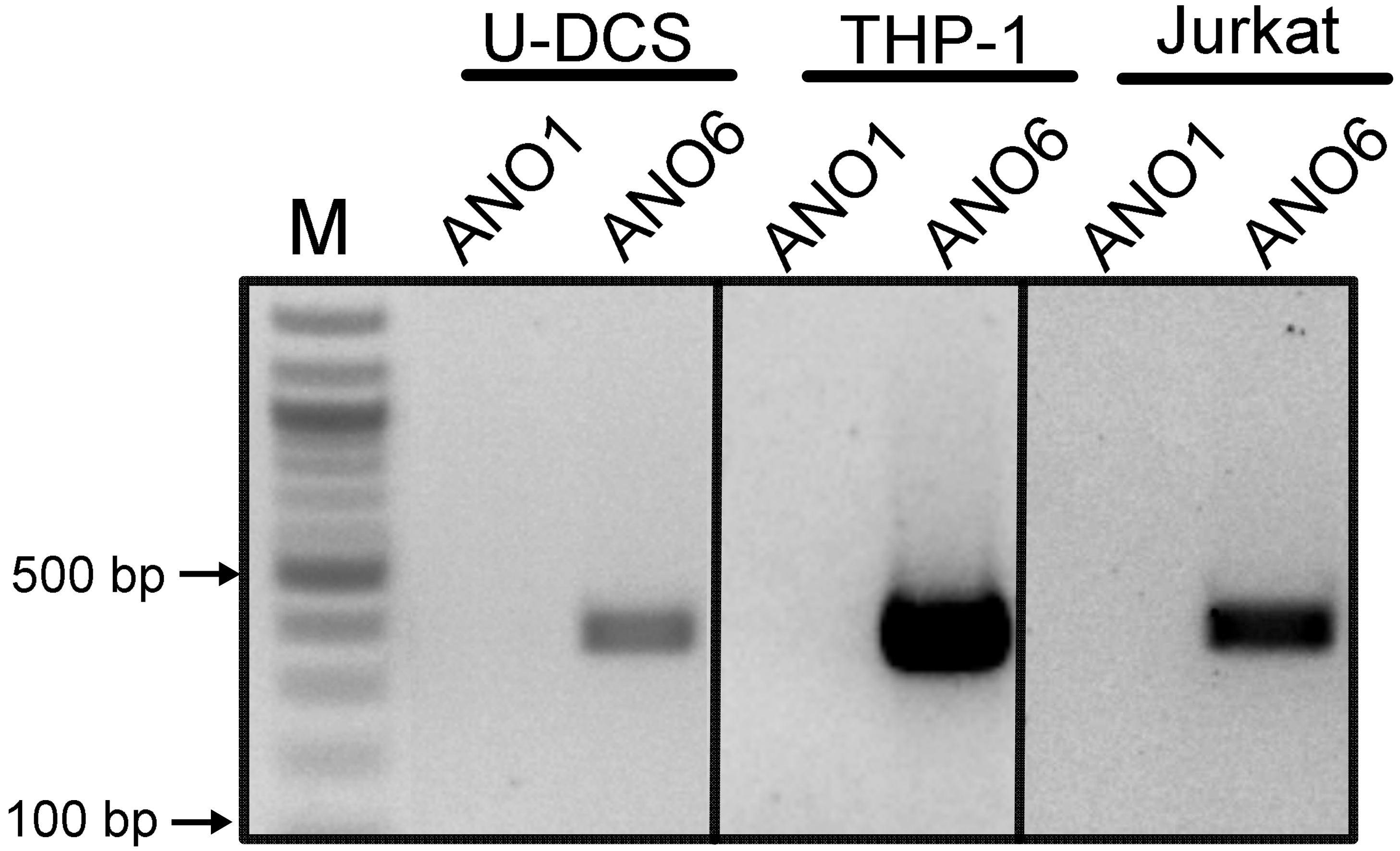

Supplementary Figure 3. *Expression of ANO1 and ANO6 in immune cells.* RT-PCR analysis of expression of ANO1 and ANO6 in human U-DCS dendritic cells, THP-1 macrophages, and Jurkat T-cells.
