# Supplementary figures and images for: Niclosamide, but not ivermectin, inhibits anoctamin 1 and 6 and attenuates inflammation of the respiratory tract
Source: Pflugers Arch. 2023 Nov 18;476(2):211–27. doi: 10.1007/s00424-023-02878-w (PMC10791962; doi:10.1007/s00424-023-02878-w)

Fig.3B

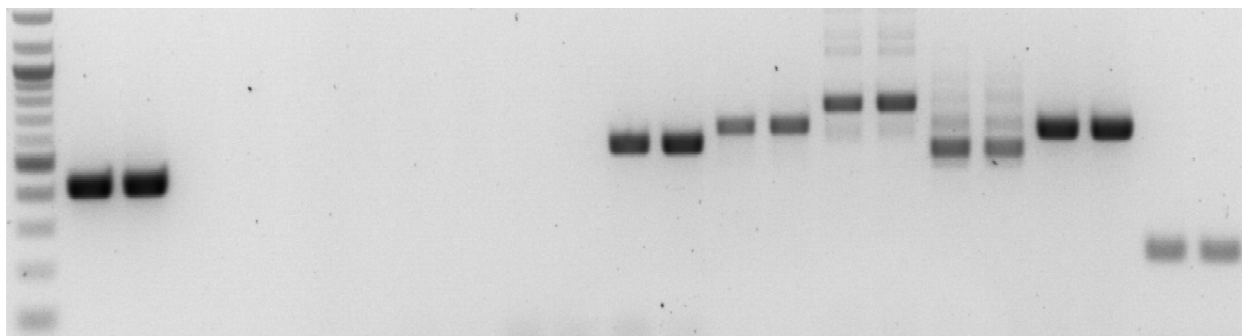

Fig.4C

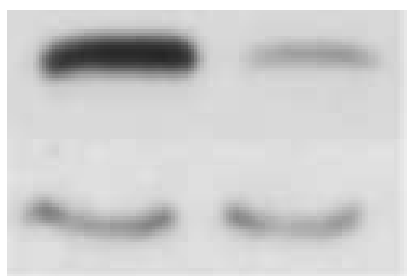

Fig.5A

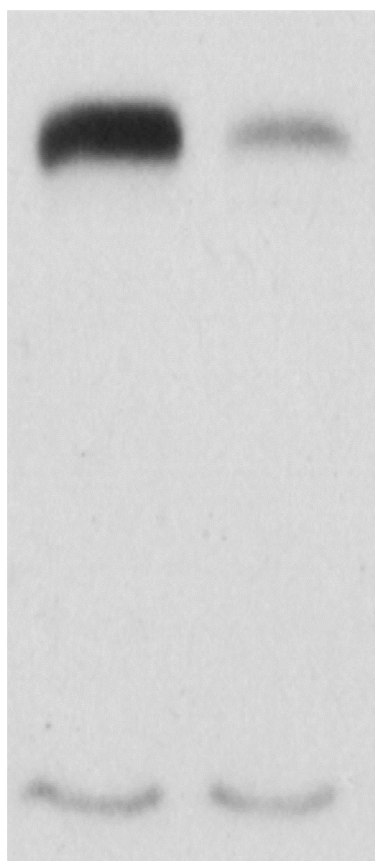

Fig.5D

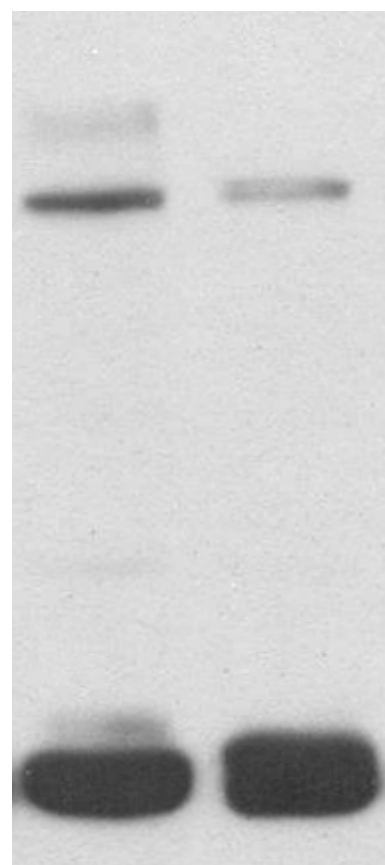

Fig.S4

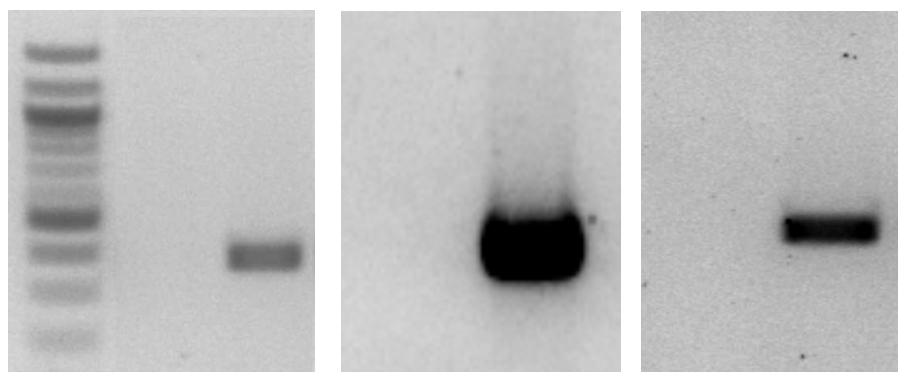

Supplement: Supplementary file 4 — Supplementary file4 (PDF 1410 KB) [file 424_2023_2878_MOESM4_ESM.pdf]
